# Supplementary material for: Vitamin C epigenetically controls osteogenesis and bone mineralization
Source: Nat Commun. 2022 Oct 6;13:5883. doi: 10.1038/s41467-022-32915-8 (PMC9537512; doi:10.1038/s41467-022-32915-8)

SUPPLEMENTARY INFORMATION

Vitamin C epigenetically controls osteogenesis and bone mineralization

Roman Thaler[1,2,3] *, Farzaneh Khani[1], Ines Sturmlechner[4], Sharareh S. Dehghani[1], Janet M. Denbeigh[1], Xianhu Zhou[1], Oksana Pichurin[1], Amel Dudakovic[1,2], Sofia S. Jerez[1,2], Jian Zhong[5], Jeong-Heon Lee[2,5], Ramesh Natarajan[6], Ivo Kalajzic[7], Yong-hui Jiang[8], David R. Deyle[9], Eleftherios P. Paschalis[10], Barbara M. Misof[10], Tamas Ordog[5,11], Andre J. van Wijnen[12] *

[1] Department of Orthopedic Surgery, Mayo Clinic, Rochester, MN, USA, [2] Department of Biochemistry & Molecular Biology, Mayo Clinic, Rochester, MN, USA, [3] Center for Regenerative Medicine, Mayo Clinic, Rochester, MN, USA, [4] Departments of Pediatric and Adolescent Medicine, Mayo Clinic, Rochester, MN, USA, [5] Epigenomics Program, Center for Individualized Medicine, Mayo Clinic, Rochester, MN, USA, [6] Department of Internal Medicine, Virginia Commonwealth University, Richmond, VA, USA, [7] Department of Reconstructive Sciences, UConn Health, Farmington, CT, USA, [8] Department of Genetics, Neuroscience, and Pediatrics, Yale University School of Medicine, New Haven, CT, USA, [9] Department of Clinical Genomics, Mayo Clinic, Rochester, MN, USA, [10] Ludwig Boltzmann Institute of Osteology at Hanusch Hospital of OEGK and AUVA, Trauma Centre Meidling, 1st Med. Dept. Hanusch Hospital, Vienna, Austria, [11] Department of Physiology nd Biomedical Engineering and Division of Gastroenterology and Hepatology, Department of Medicine, Mayo Clinic, Rochester, MN, USA, [12] Department of Biochemistry, University of Vermont, Burlington, VT, USA. *Correspondence: Thaler.Roman@mayo.edu, andre.vanwijnen@uvm.edu

TABLE OF CONTENTS

Supplementary Figures 1-17
Supplementary Tables 1-2
Uncropped blots for Supplementary Figures

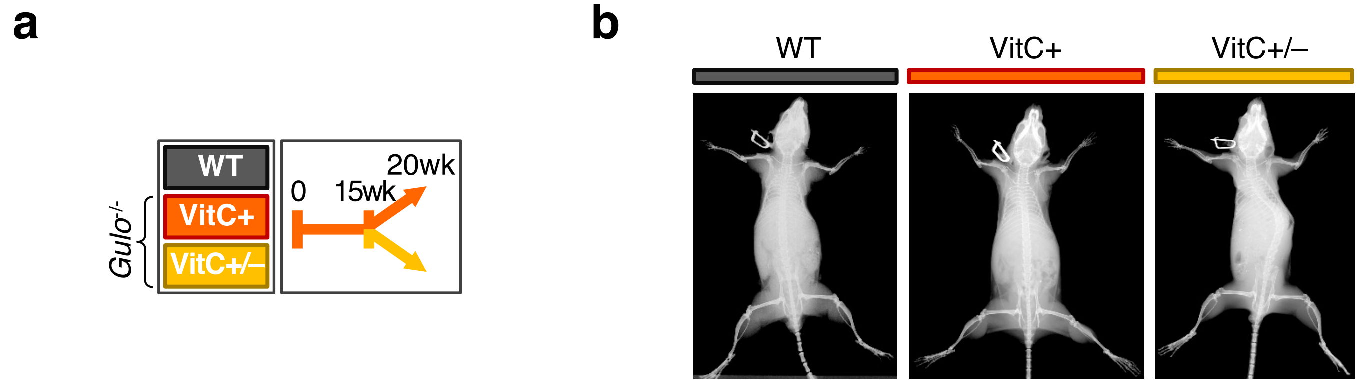

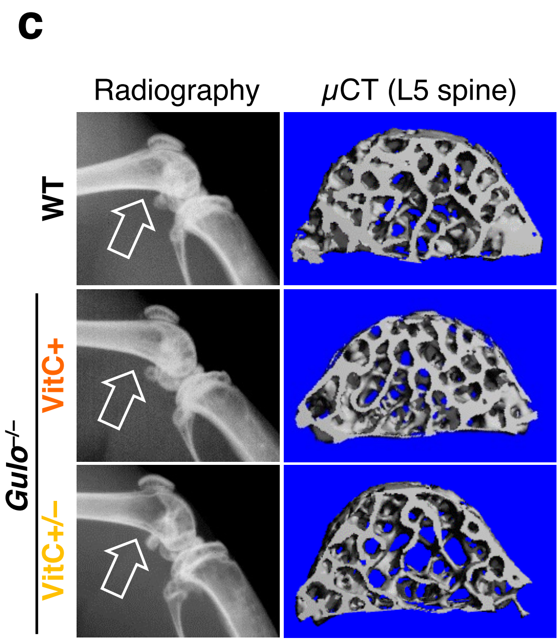

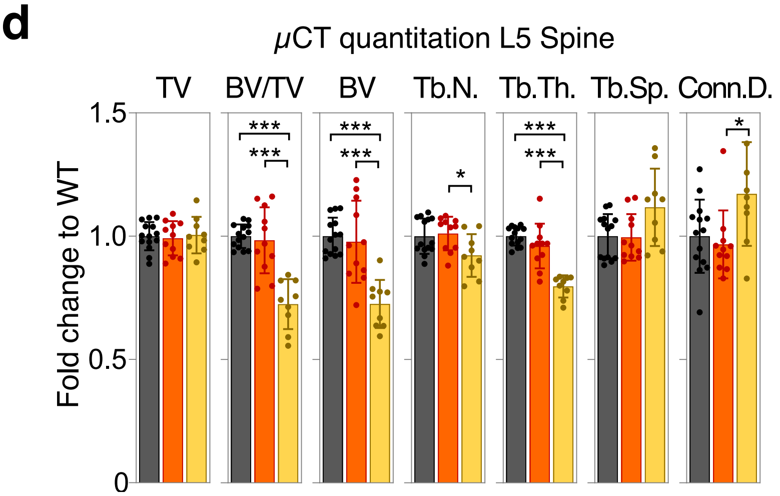

	BV	TV	BV/TV	Tb.N.
**WT**	1.00 (±0.07)	1.00 (±0.06)	1.00 (±0.05)	1.00 (±0.07)
**VitC+**	0.98 (±0.17)	0.99 (±0.07)	0.98 (±0.13)	1.01 (±0.07)
**VitC+/–**	0.72 (±0.10)	1.00 (±0.07)	0.72 (±0.10)	0.92 (±0.08)

	Tb.Th	Tb.Sp	Conn.D.	SMI
**WT**	1.00 (±0.04)	1.00 (±0.09)	1.00 (±0.15)	1.00 (±0.64)
**VitC+**	0.96 (±0.09)	0.99 (±0.09)	0.97 (±0.14)	1.07 (±1.98)
**VitC+/–**	0.78 (±0.04)	1.12 (±0.16)	1.17 (±0.21)	-1.64 (±1.20)

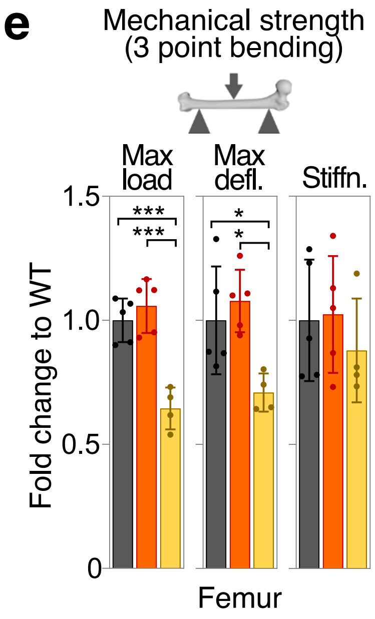

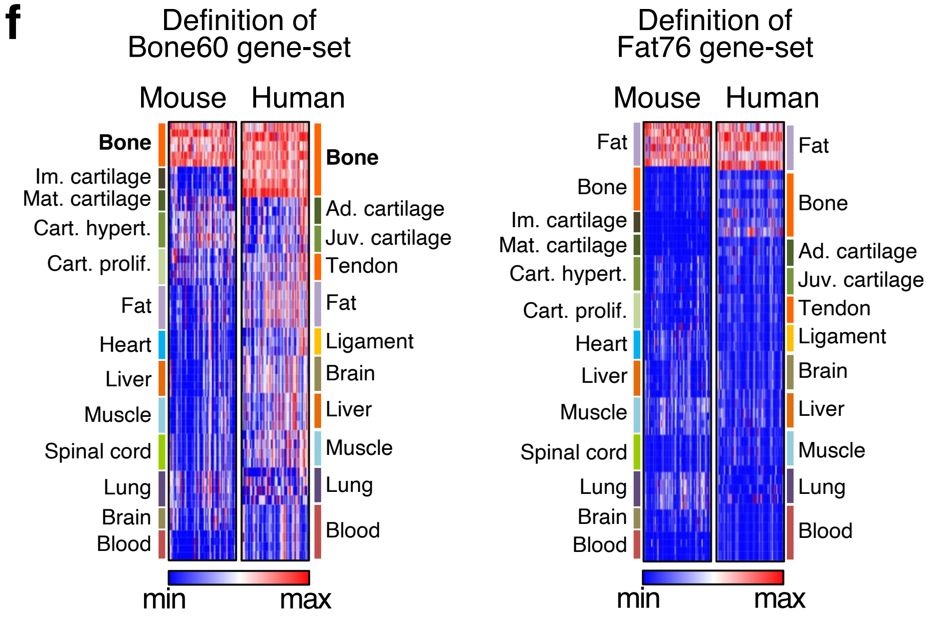

Supplementary Figure 1: Vitamin C deficiency impairs bone volume and bone quality in *Gulo* knockout mice. a Experimental setup. **b** Whole body radiographic pictures (**b**) as well as Femur/tibia radiography and L5 spine micro-computed tomography (µCT) images (**c**) from *Gulo*$^{-/-}$ mice supplemented with VitC (VitC+) for 20 weeks, or VitC-supplemented for 15 weeks followed by VitC withdrawal for 5 weeks (VitC+/-). Wild type (WT) mice were used as additional control. **d** graphical illustration and detailed listing of µCT analysis for L5 spine; BV, bone volume; TV, total volume; Tb.N., trabecular number; Tn.Th., trabecular thickness; Tb.Sp., trabecular separation; Conn.D., connectivity density; SMI, structure model index. **e** Three-point bending test of femurs in groups indicated in (**a**). Max defl., maximum deflection; Stiffn., stiffness. **f** Gene expression heatmap showing tissue-selective Bone60 and Fat76 gene-sets generated by analyzing 47 human and 59 mouse RNA-seq datasets. Graphs represent mean ± SD. *p<0.05; ***p<0.001; One-way ANOVA with Tukey's multiple comparison tests (**d,e**). DESeq-2 was used for the analysis of (**f**) using standard settings and batch correction. In (**d**), n=14 for WT, n=11 for VitC+ and n=9 for VitC+/-; in (**e**), n=5 for WT & VitC+ and n=4 for VitC+/-; all samples were derived from biologically independent animals. Source data as well as exact p=values for all comparisons in (**d**,**e**) are provided in the Source Data File.

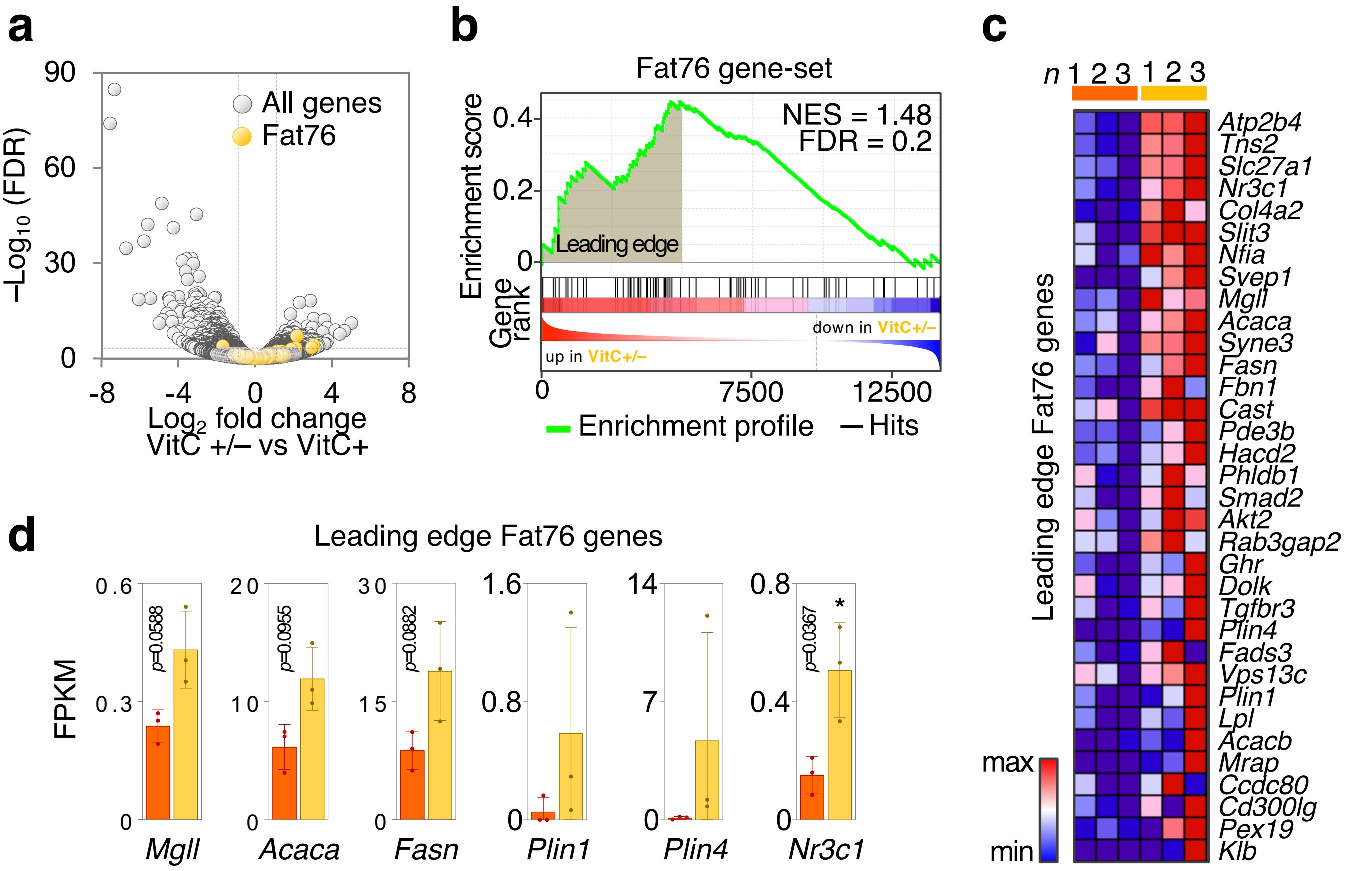

Supplementary Figure 2: Vitamin C deprivation does not diminish the expression of adipogenic marker genes. a Volcano plot and **b** GSEA analysis of the Fat76 gene-set in VitC+/- versus VitC+ RNA-seq data from *Gulo*$^{-/-}$ femurs; NES, normalized enrichment score; FDR, false discovery rate. **c,d** Heatmap and gene expression bar charts depict leading-edge genes. Bar graphs represent mean ± SD. *p<0.05; FDR adjusted Wald test (**a**), FDR-adjusted two-tailed, unpaired t-tests (**d**); *N*=3 per group from biologically independent animals (**d**). Source data are provided as a Source Data File.

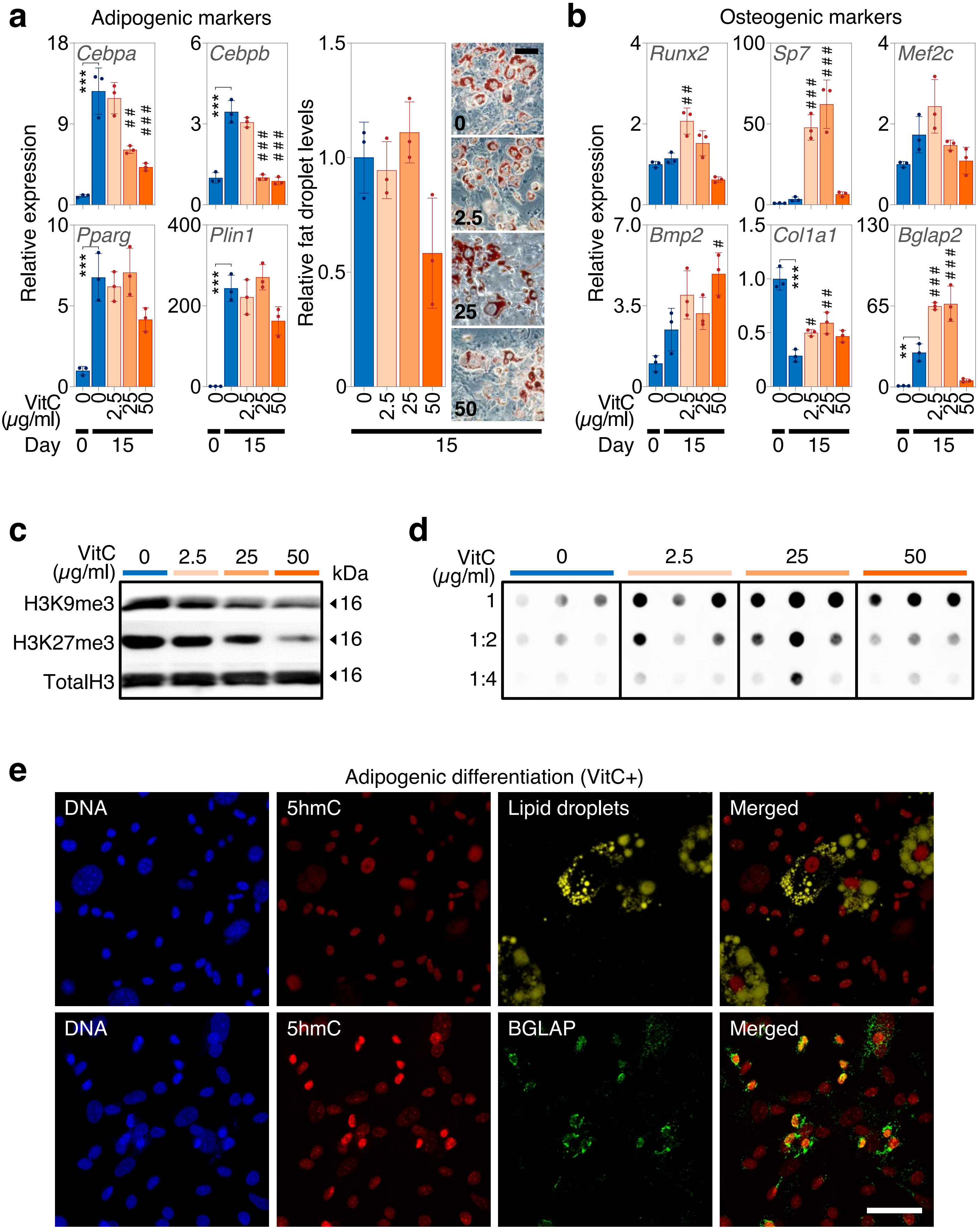

a
Adipogenic markers
Cebpa
Cebpb
Pparg
Plin1
Relative expression
Relative fat droplet levels
VitC (µg/ml)
Day
0
15
b
Osteogenic markers
Runx2
Sp7
Mef2c
Bmp2
Col1a1
Bglap2
c
VitC (µg/ml)
0
2.5
25
50
kDa
H3K9me3
H3K27me3
TotalH3
16
d
VitC (µg/ml)
1
1:2
1:4
e
Adipogenic differentiation (VitC+)
DNA
5hmC
Lipid droplets
Merged
BGLAP

Supplementary Figure 3: Adipose lineage differentiation does not require Vitamin C. **a** Adipogenic markers gene expression and fat droplet levels in mouse BMSCs differentiated to adipocytes in the presence or absence of varying VitC concentrations. **b** Osteogenic markers gene expression in the same cultures as in (**a**). **c** H3K9me3 and H3K27me3 western blot. **d** 5hmC dot blot in adipocyte cultures at D15 of differentiation with or without varying concentrations of VitC. **e** Immunofluorescence of 5hmC and BGLAP as wells as *LipidSpot*-fluorescent fat droplet detection in VitC (25 μg/ml) - treated adipocyte cell cultures differentiated from BMSCs at d15. Note that BGLAP-positive cells show increased 5hmC levels. In **a** and **e** scale bar represents 50 μm. Bar graphs represent mean ± SD, **$p<0.01$; ***$p<0.001$ comparing groups to VitC 0 at d0; #$p<0.05$; ##$p<0.01$; ###$p<0.001$ comparing groups to VitC 0 at d15; one-way ANOVA with Tukey's multiple comparison tests; $n=3$ per group from cells derived from biologically independent animals (**a**,**b**). Source data as well as exact p=values for all comparisons in (**a**,**b**) are provided in the Source Data File.

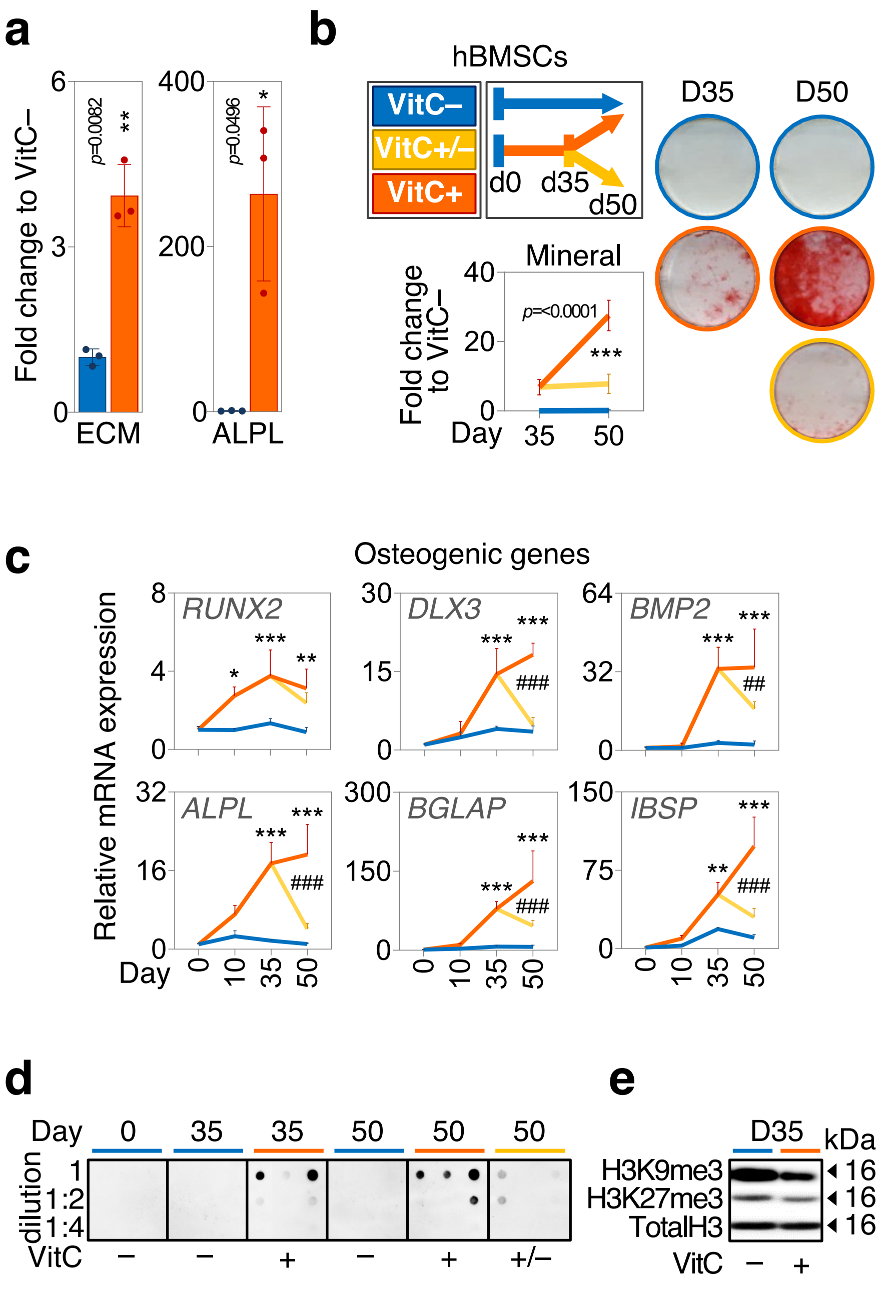

Supplementary Figure 4: Vitamin C controls osteogenic differentiation of human BMSCs. a Extracellular matrix (ECM) deposition and ALPL activity at day 10 in human BMSCs with or without VitC. **b** Experimental setup for (**b-d**) as well as mineral deposition with or without VitC at day 35 and after removal of VitC from day 35 to day 50. **c** mRNA expression of osteoblast markers in hBMSCs with or without VitC, or cultures in which VitC was removed starting at day 35 (VitC+/-). **d** 5hmC dot blot in hBMSCs with or without VitC and at day 35 and after removal of VitC from day 35 to day 50. **e** H3K9me3 and H3K27me3 western blot in hBMSCs with or without VitC at day 35. Graphs represent mean ± SD, *$p<0.05$; **$p<0.01$; ***$p<0.001$. Paired two-tailed *t*-test in (**a**), two-way ANOVA with Tukey's multiple comparison tests in (**b,c**); in (**c**) * marked significances represent comparisons between VitC+ vs VitC- at the indicated time point and # significances represent comparisons between D50 VitC+ vs D50 VitC+/-. *N*=3 per group from cells derived from biologically independent donors (**a-c**). Source data as well as exact *p*=values for all comparisons in (**c**) are provided in the Source Data File.

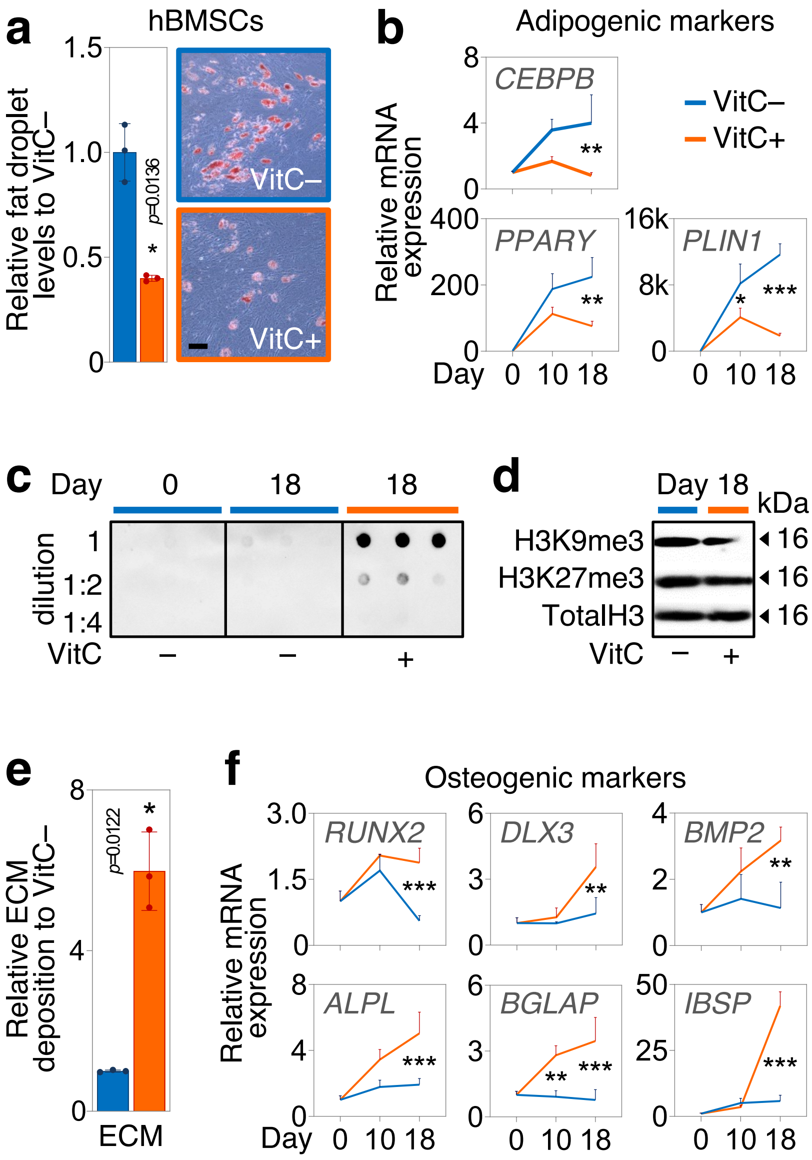

Supplementary Figure 5: Vitamin C is dispensable for adipogenic differentiation of human BMSCs. a Fat droplet levels at day 18 in human BMSCs differentiated to adipocytes with or without VitC, scalebar 100 µm. **b** mRNA expression of adipogenic genes in hBMSCs differentiated to adipocytes with or without VitC. **c,d** 5hmC dot blot and H3K9me3 and H3K27me3 western blot in adipocytes differentiated from hBMSCs with or without VitC **e** ECM deposition in to adipocytes differentiating hBMSCs treated with or without VitC at day 10. **f** mRNA expression of osteogenic genes in hBMSCs differentiated to adipocytes with or without VitC. Graphs represent mean ± SD, **p*<0.05; ***p*<0.01; ****p*<0.001. Paired two-tailed *t*-test in (**a**,**e**), two-way ANOVA with Tukey's multiple comparison tests showing direct comparisons for each time point in (**b**,**f**). *N*=3 per group from cells derived from biologically independent donors (**a**,**b**,**e**,**f**). Source data as well as exact *p*=values for all comparisons in (**b**,**f**) are provided in the Source Data File.

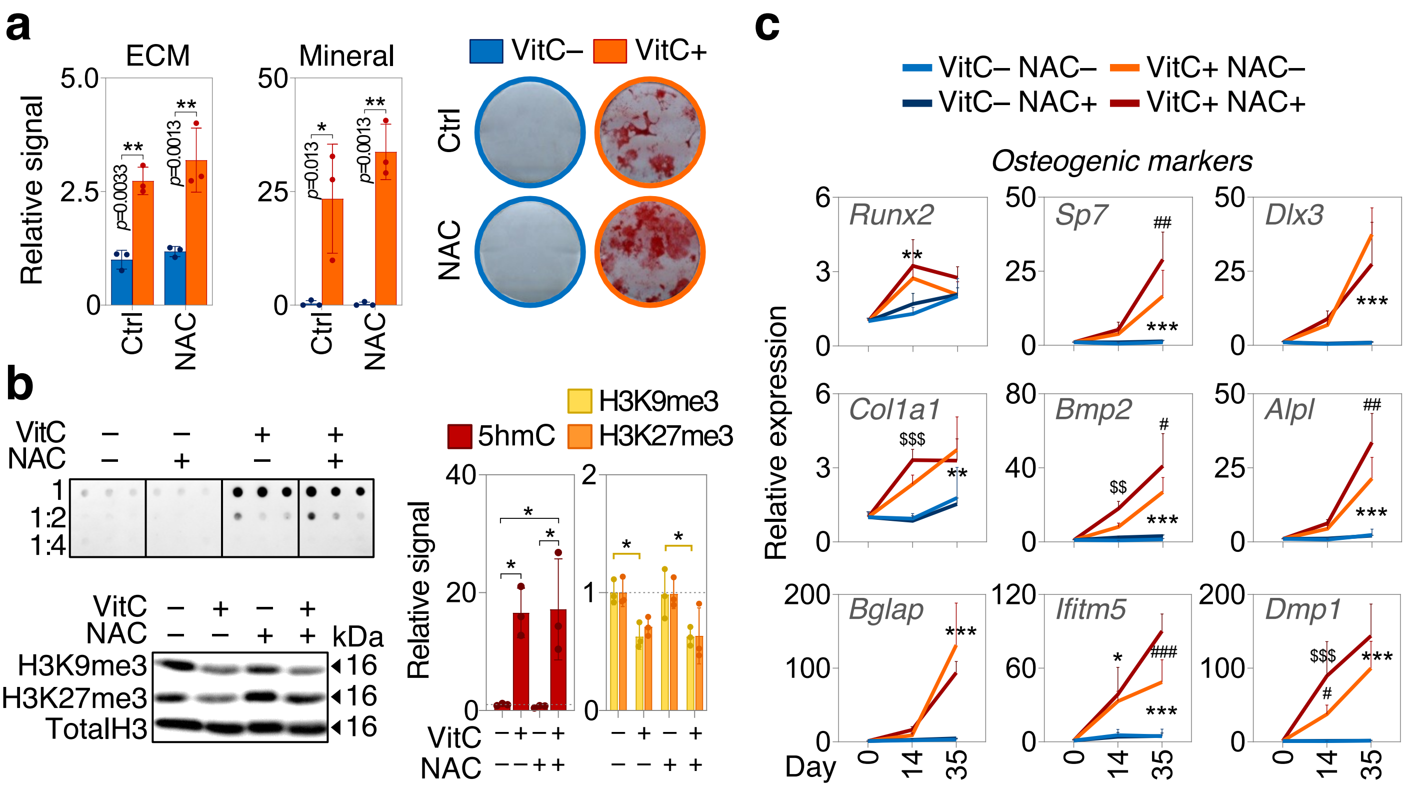

Supplementary Figure 6: The antioxidant function of Vitamin C is independent from its pro-osteogenic role. **a** ECM deposition at day 15 and ECM mineralization at day 35 in mBMSCs treated with or without VitC and with or without 3mM N-acetyl-cysteine (NAC). **b** Effects of 3 mM NAC on 5hmC levels as shown by dot blot as well as on H3K9me3 and H3K27me3 amounts as shown by western blot including quantification by relative densitometry of the blots. **c** mRNA expression of osteogenic markers in mBMSCs exposed to 3 mM NAC during osteogenic differentiation. Graphs represent mean ± SD, *p<0.05; **p<0.01; ***p<0.001; #p<0.05; ##p<0.01; ###p<0.001; $$$p$<0.01; $$$$p$<0.001. In (**c**) * tests VitC- NAC- vs VitC+ NAC-; # tests VitC+ NAC- vs VitC+ NAC+; $ tests VitC- NAC- vs VitC+ NAC+ at the corresponding time points. Two-way ANOVA (**a**,**b** histones, **c**) or one-way ANOVA (**b**, 5hmC) with Tukey's multiple comparison tests. *N*=3 per group from cells derived from biologically independent animals (**a**-**c**). Source data as well as exact *p*=values for all comparisons in (**b**,**c**) are provided in the Source Data File.

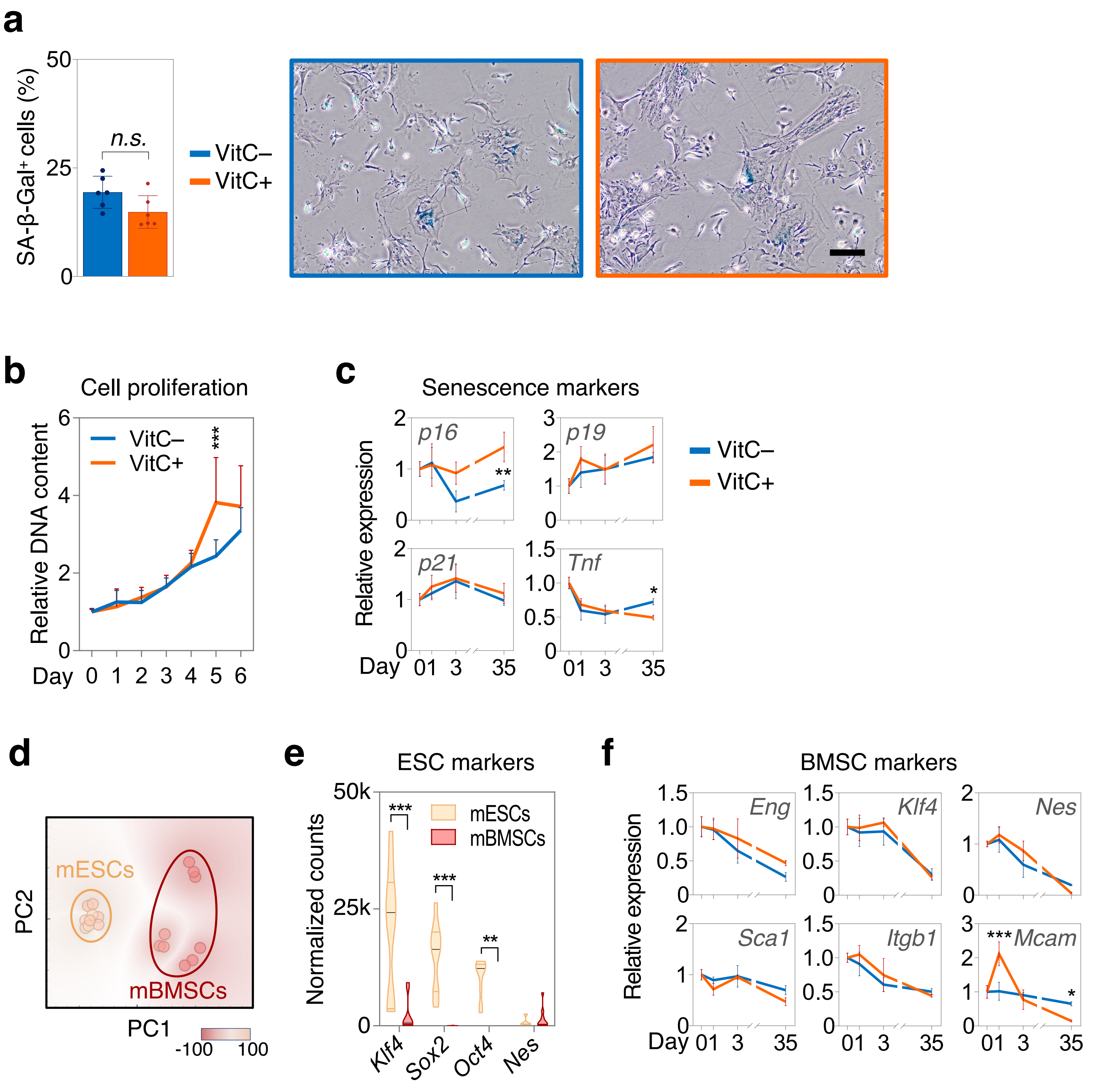

Supplementary Figure 7: Cellular senescence and stemness markers of BMSCs are largely unaffected by Vitamin C. a Proportion of mBMSCs positive for senescence-associated β-Galactosidase (SA-β-Gal) with or without VitC at day 3 and representative images; scale bar 50 μm. **b** Cell proliferation profile of mBMSCs treated with or without VitC. **c** mRNA expression of senescence markers in mBMSCs treated with or without VitC. **d** Principal component (PC) analysis comparing global mRNA expression data (RNA-Seq) from mouse embryonic stem cells (mESCs) and mBMSCs. **e** RNA-Seq expression of ESCs markers in mESCs vs mBMSCs. **f** mRNA expression of BMSC markers in mBMSCs treated with or without VitC. Graphs represent mean ± SD, *p<0.05; **p<0.01; ***p<0.001. Two-way ANOVA with Sidak's multiple comparison tests for (**b,c,e,f**) and unpaired t test for (**a**). N=6 (a) & n=8 (b) per group from independent experiments, n=3 (**c**,**f**) & n=9 (**e**) per group from biological independent animals. Source data as well as exact p=values for all comparisons in (**a-c**,**e**,**f**) are provided in the Source Data File.

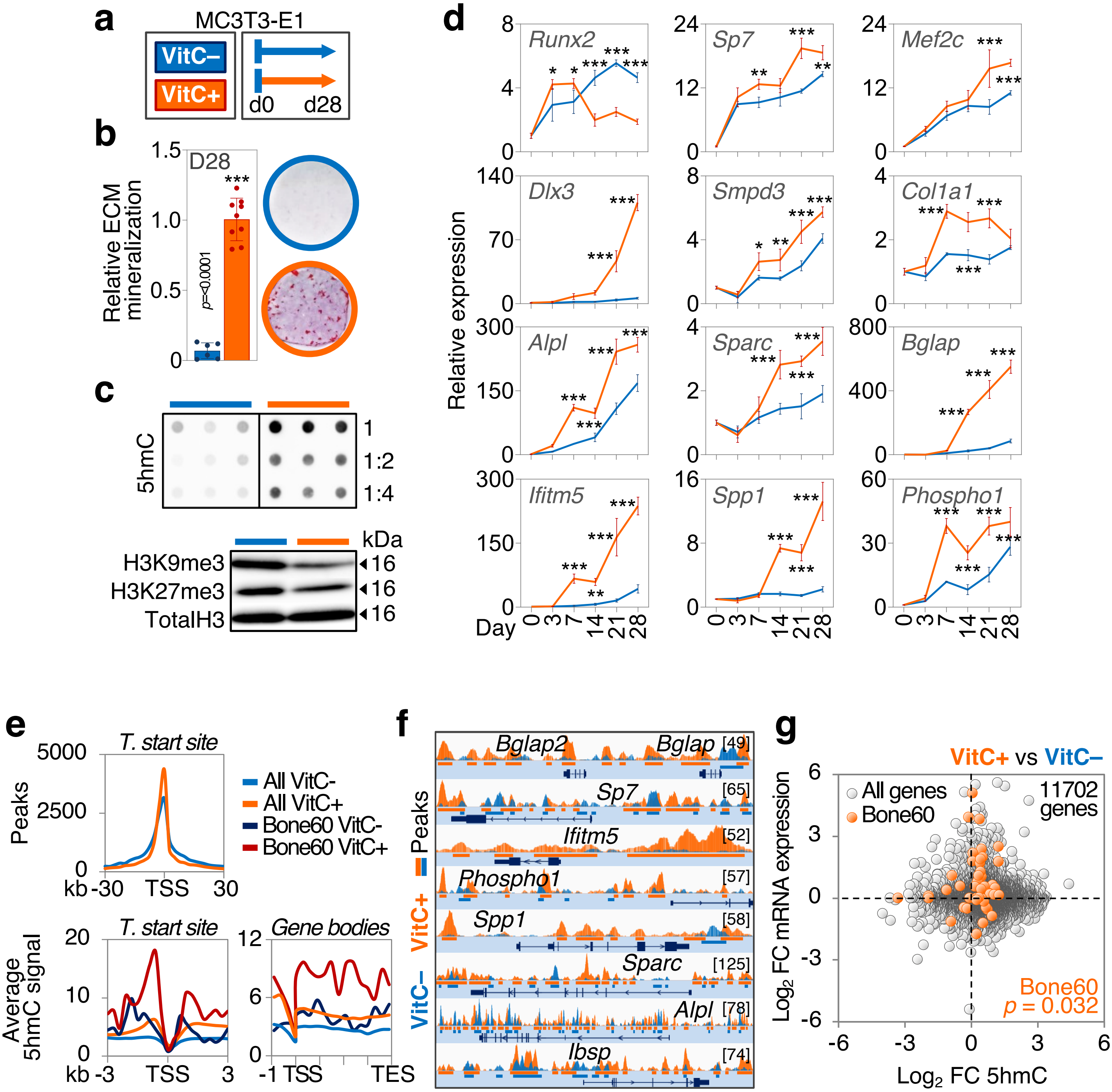

Supplementary Figure 8: Differentiation of MC3T3-E1 osteoblasts with Vitamin C involves chromatin relaxation and selective hydroxymethylation of osteogenic loci. a Experimental setup. **b** Extracellular matrix (ECM) mineralization. **c** 5hmC dot blot as well as H3K9me3 and H3K27me3 western blot during MC3T3-E1 differentiation with or without VitC. **d** Osteogenic gene expression in VitC-untreated or -treated MC3T3-E1 osteoblasts. **e** hMeDIP-seq analysis of MC3T3-E1 showing 5hmC peak number around transcriptional start sites (TSS), average 5hmC signal around TSS or in gene bodies for all genes or for the Bone60 gene-set; TES, transcriptional end site. **f** Overlaid 5hmC peak occupancy comparing VitC- and VitC+ groups near Bone60 genes; [value]=max peak scale. **g** Correlation between gene expression and 5hmC occupancy (TSS +/-30 kb) in VitC+ vs VitC- treated cells; FC, fold change. Bar and line graphs represent mean ± SD; *p<0.05; **p<0.01; ***p<0.001. Unpaired two-sided t-test (**b**), two-way ANOVA with Sidak's multiple comparison tests (**d**, groups were compared at each time point), two-sided Fishers exact test (**g**). N=9 for VitC+ and n= 6 for VitC- in (**b**), n=3 in (**d**); all data is derived from independent experiments. Source data as well as exact p=values for all comparisons in (**d**) are provided in the Source Data File.

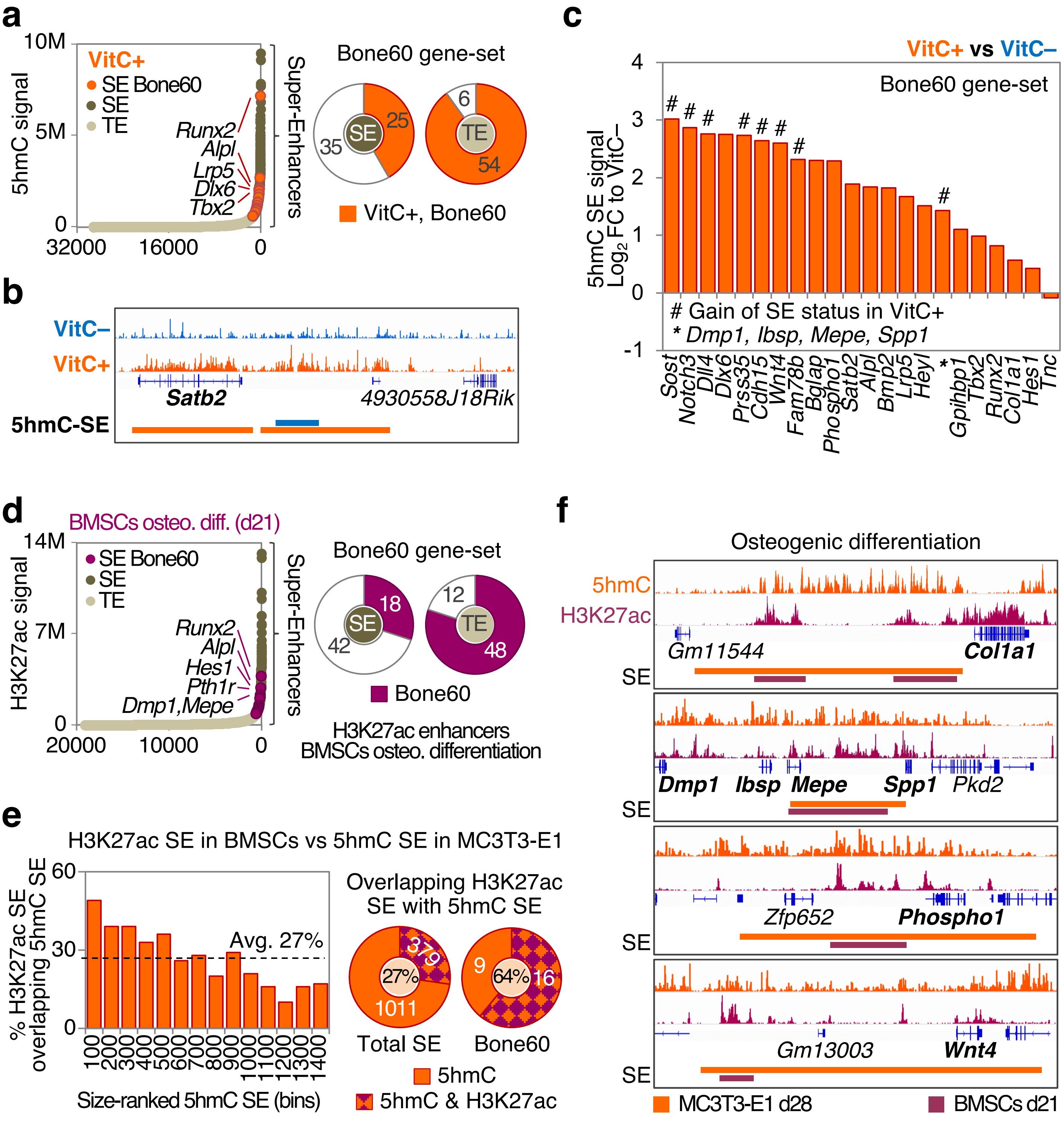

Supplementary Figure 9: Vitamin C-dependent, distal 5hmC peaks in MC3T3-E1 co-localize with H3K27ac-marked super-enhancers in osteoblasts derived from mouse BMSCs. **a** Super-enhancer (SE) based clustering of 5hmC peaks with *Runx2* as the highest ranking Bone60 gene and number of Bone60 genes associated with 5hmC-SE and 5hmC-typical enhancer (TE). **b** Example of 5hmC-SE. **c** New formation of 5hmC-SE and ranked increase in 5hmC signal at 5hmC-SE in VitC treated vs untreated cells. **d** H3K27ac SE analysis in osteogenic differentiated BMSCs with *Runx2* as the highest ranking Bone60 gene and number of H3K27ac enhancers and SE associated with Bone60 genes. **e** Amount of 5hmC SE from VitC+ MC3T3-E1 cells (D28) overlapping with H3K27ac SE from osteogenic BMSCs and count of 5hmC SE-positive Bone60 genes in MC3T3-E1 cells with overlapping H3K27ac SE in BMSCs. **f** Representative selection of Bone60 genes comparing 5hmC SE and H3K27ac SE in differentiated osteoblasts. Source data are provided as a Source Data File.

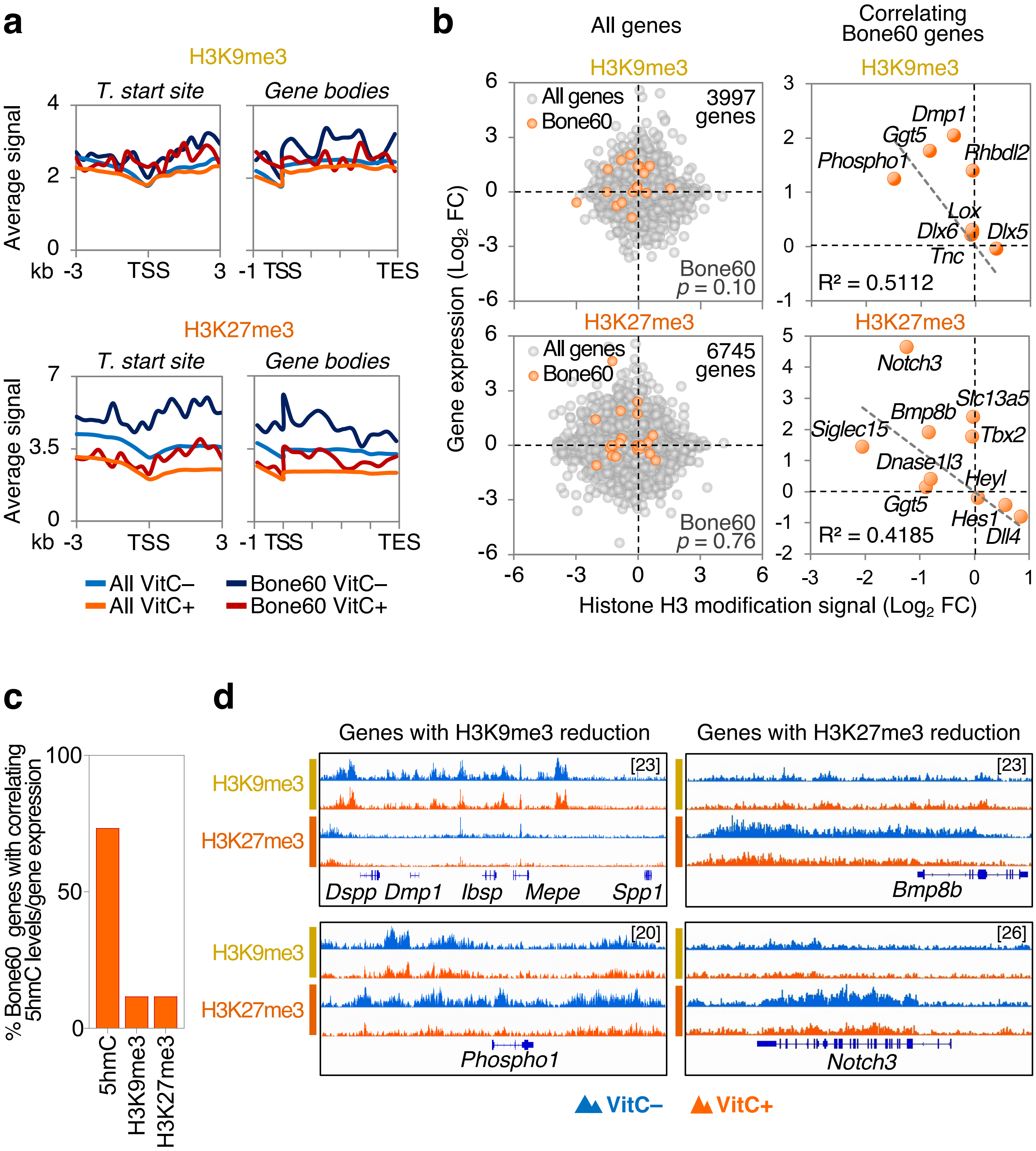

Supplementary Figure 10: Vitamin C affects selective Histone demethylation patterns at a subset of osteogenic genes. **a** H3K9me3 and H3K27me3 ChIP-seq analysis of MC3T3-E1 cells treated with or without VitC showing average peak signals around transcription start site (TSS) or in gene bodies for all genes or for the Bone60 gene-set; TES, transcription end site. **b** Correlation between gene expression and H3K9me3 or H3K27me3 occupancy (TSS +/-30 kb) showing all genes and correlating Bone60 genes in VitC+ vs VitC- treated cells; FC, fold change. **c** Percentage of Bone60 genes where VitC dependent levels of shown epigenetic marks (TSS +/-30 kb) correlate with their mRNA expression. **d** H3K9me3 and H3K27me3 peak occupancy comparing VitC- and VitC+ groups near selected Bone60 genes; [value]=max peak scale. Two-sided Fishers exact test (**b**). Source data are provided as a Source Data File.

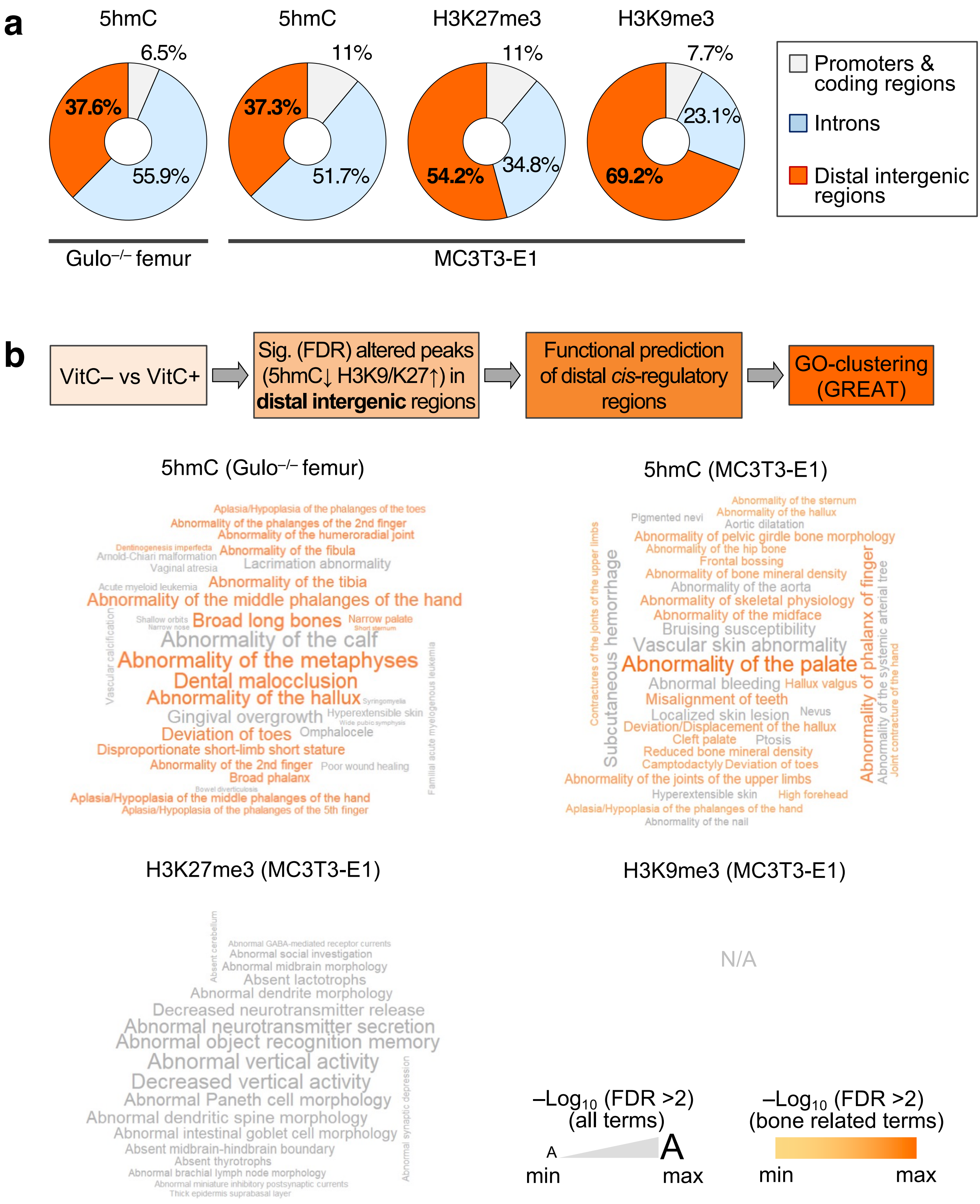

Supplementary Figure 11: Vitamin C-sensitive, distal intergenic 5hmC peaks associate with genes linked to skeletal phenotypes. **a** Genomewide peak distribution for shown epigenetic marks in VitC treated bone tissue and MC3T3-E1 osteoblasts. **b** Analysis pathway and word-cloud representation of GREAT-mediated GO-phenotypes for shown marks and substrates. FDR, false discovery rate. Source data are provided as a Source Data File.

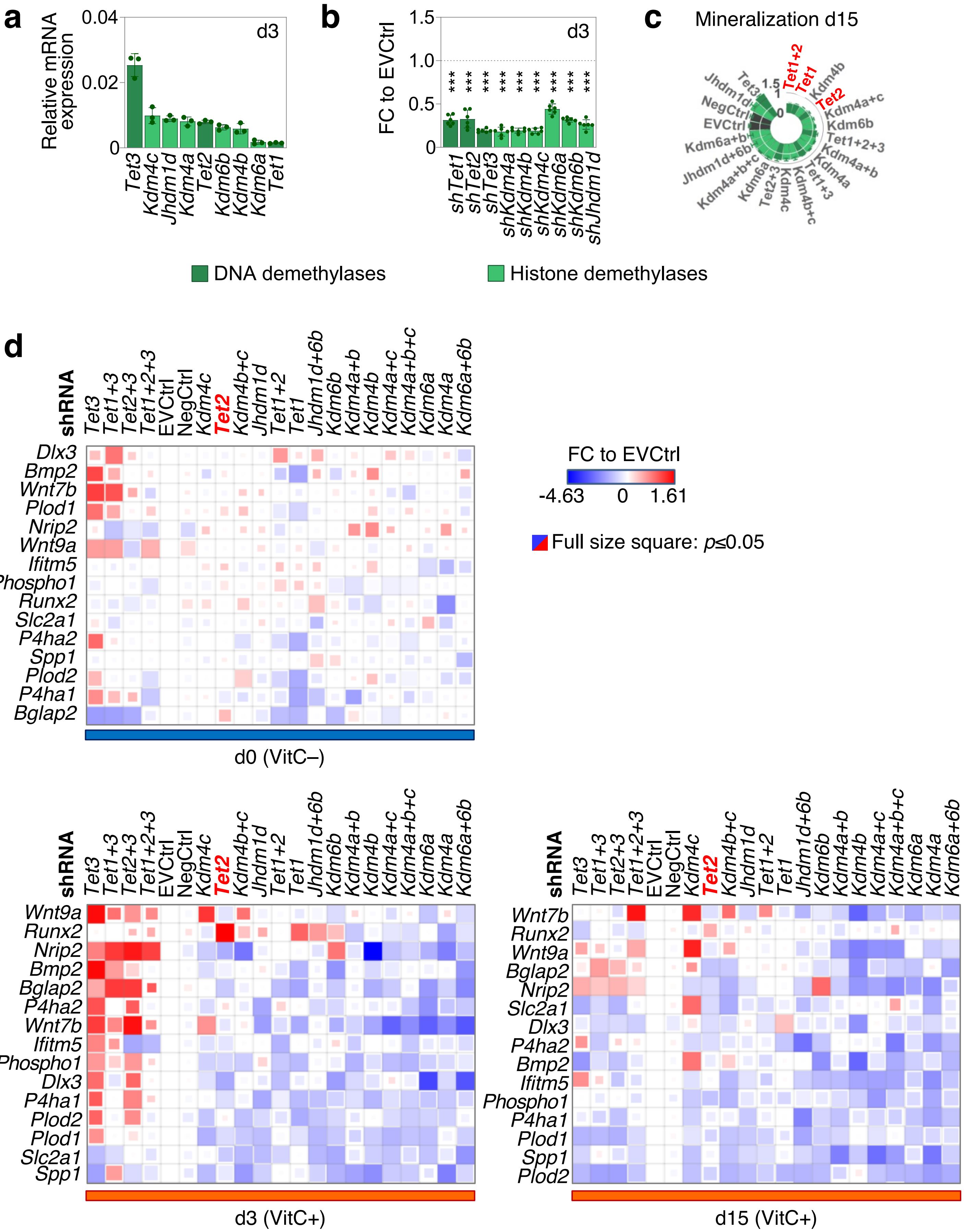
a
Relative mRNA expression
0.04
0.02
0
d3
Tet3
Kdm4c
Jhdm1d
Kdm4a
Tet2
Kdm6b
Kdm4b
Kdm6a
Tet1
b
FC to EVCtrl
1.5
1.0
0.5
0
d3
***
shTet1
shTet2
shTet3
shKdm4a
shKdm4b
shKdm4c
shKdm6a
shKdm6b
shJhdm1d
c
Mineralization d15
Tet1+2
Tet1
Tet2
DNA demethylases
Histone demethylases
d
shRNA
FC to EVCtrl
-4.63
0
1.61
Full size square: p≤0.05
d0 (VitC–)
d3 (VitC+)
d15 (VitC+)

Supplementary Figure 12: Histone demethylases and DNA hydroxymethylases are required during late stages of osteogenic differentiation. a Ranked basal hydroxylases gene expression from MLO-A5 pre-osteocytes as shown by rt-qPCR data. **b** shRNA-mediated knockdown efficiencies at day 3. **c** Ranked ECM mineralization after hydroxylases shRNA knockdowns at day 15. **d** Two-way ranked osteoblastic gene expression after hydroxylases shRNA knockdowns in differentiating MLO-A5 cells. Bar graphs represent mean ± SD; ***$p<0.001$. One-way ANOVA with Dunnett's multiple comparison tests (**b**). Samples were compared to EVCtrl (**b,d**). $N=3$ (**a**,**c**), $n=3$/tested shRNA (**b**), all from biological independent experiments. FC; fold change; EV, empty vector. Source data are provided as a Source Data File.

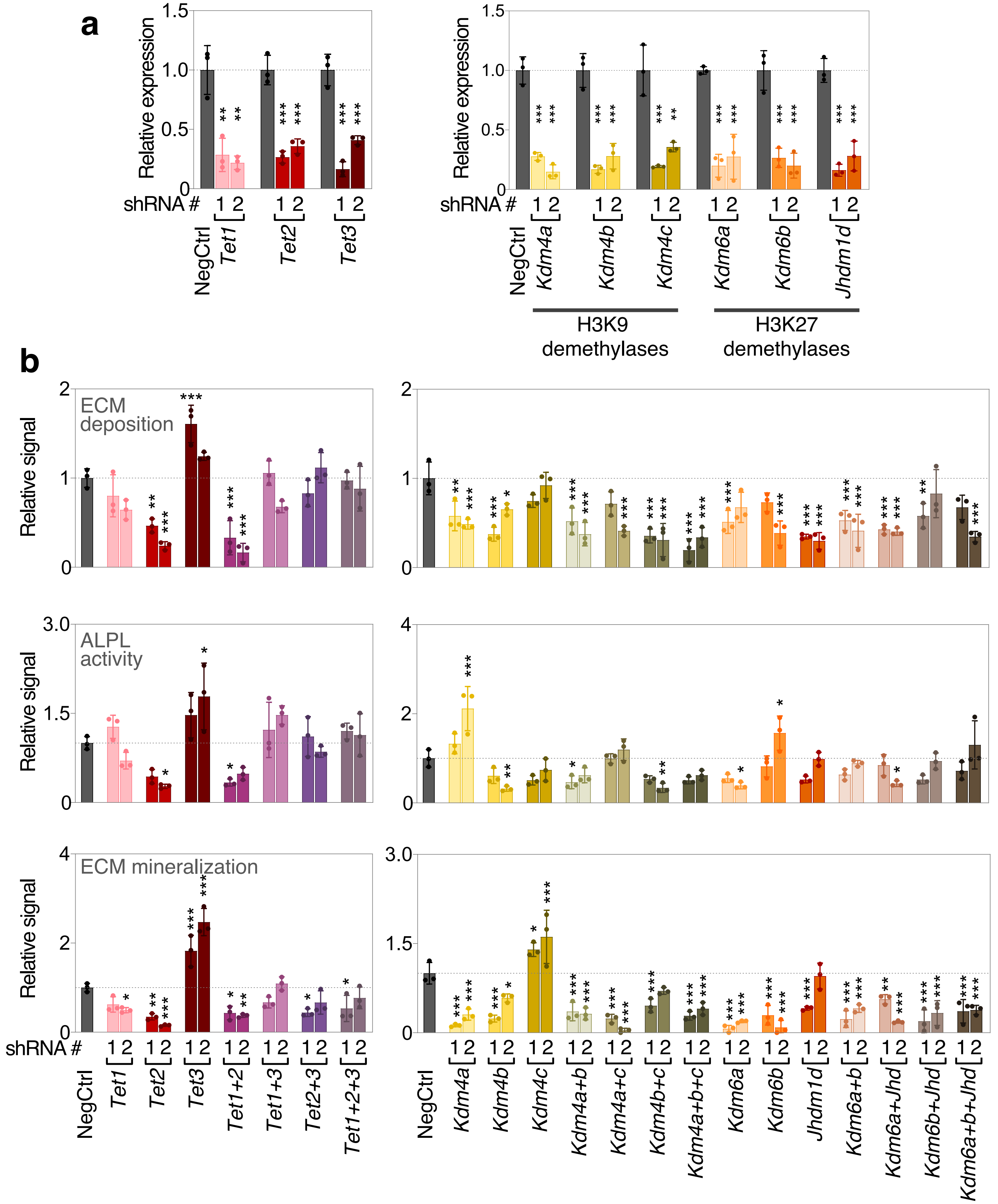

Supplementary Figure 13: Lack of Vitamin C-dependent epigenetic modulators constrains osteogenic differentiation to varying severities in mBMSCs. **a** Knockdown efficiencies for DNA demethylases and H3K9me3 and H3K27me3 demethylases by shRNAs at day 3 of differentiation. **b** Effects of knockdowns on ECM deposition at day 10, ALPL activity at day 10 and ECM mineralization at day 35. Graphs represent mean ± SD, **p*<0.05; ***p*<0.01; ****p*<0.001. One-way ANOVA testing against NegCtrl using Dunnett's multiple comparison test. *N*=3 (**a**,**b**), all from biological independent experiments. Source data as well as exact *p*=values for all comparisons are provided in the Source Data File.

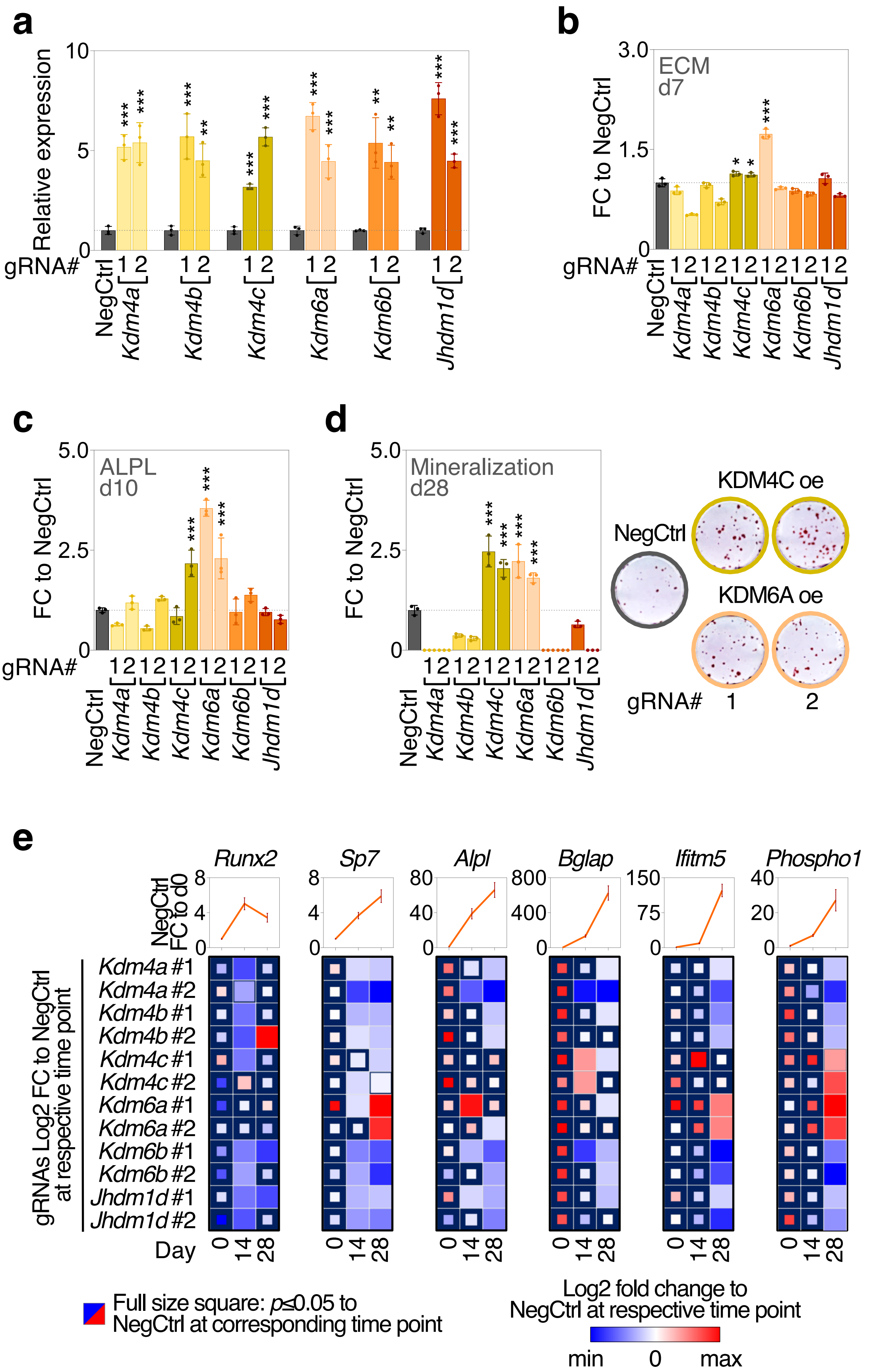
a
Relative expression
10
5
0
gRNA#
1 2
NegCtrl
Kdm4a
Kdm4b
Kdm4c
Kdm6a
Kdm6b
Jhdm1d
***
**
b
FC to NegCtrl
3.0
1.5
0
ECM
d7
c
5.0
2.5
ALPL
d10
d
Mineralization
d28
NegCtrl
KDM4C oe
KDM6A oe
gRNA# 1 2
e
Runx2
Sp7
Alpl
Bglap
Ifitm5
Phospho1
NegCtrl FC to d0
8
4
80
40
800
400
150
75
40
20
gRNAs Log2 FC to NegCtrl at respective time point
Kdm4a #1
Kdm4a #2
Kdm4b #1
Kdm4b #2
Kdm4c #1
Kdm4c #2
Kdm6a #1
Kdm6a #2
Kdm6b #1
Kdm6b #2
Jhdm1d #1
Jhdm1d #2
Day
0
14
28
Full size square: p≤0.05 to NegCtrl at corresponding time point
Log2 fold change to NegCtrl at respective time point
min
0
max

Supplementary Figure 14: Overexpression of histone demethylases facilitates selected aspects of osteoblastic differentiation. a Efficiencies of gRNAs for the endogenous overexpression of the respective demethylases at the mRNA level in MC3T3-E1 cells. **b-d** Effects of demethylases overexpression on ECM deposition (**b**), ALPL activity (**c**) and ECM mineralization (**d**). **e** mRNA expression of osteogenic markers after demethylases overexpression; line graphs on top represent mRNA expression for NegCtrls, heat maps underneath refer to fold changes to NegCtrls at respective time points. Bar graphs represent mean ± SD, *$p<0.05$; **$p<0.01$; ***$p<0.001$. One-way ANOVA with Dunnett's multiple comparison test to NegCtrl (**a-d**); two-way ANOVA with Dunnett's multiple comparison test to NegCtrl of the respective gene at corresponding time point (**e**). $N=3$ (**a-d**), all from biological independent experiments. FC, fold change. Source data as well as exact p=values for all comparisons in (**a**-**d**) are provided in the Source Data File.

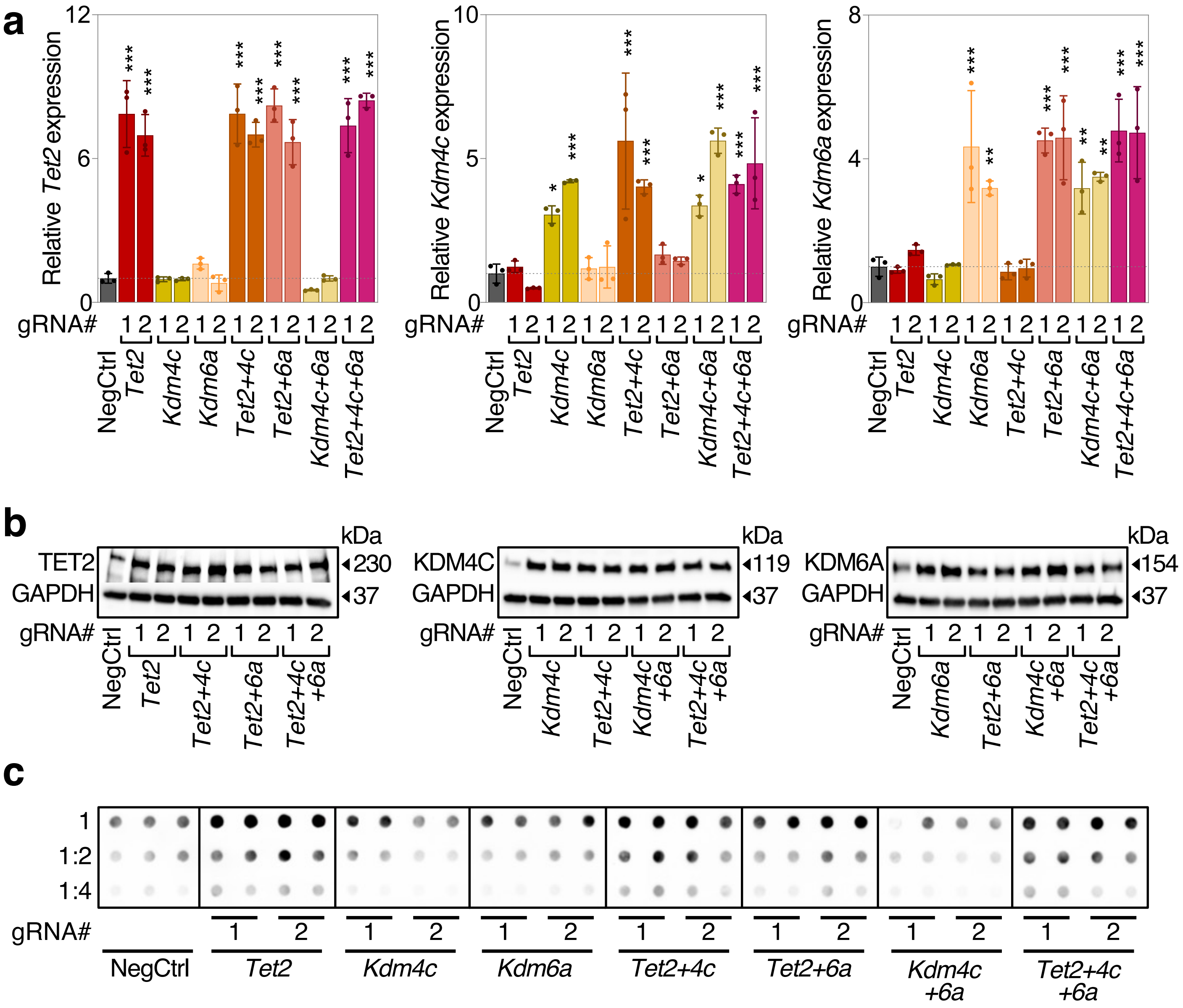

Supplementary Figure 15: Combined overexpression of DNA and histone demethylases in MC3T3-E1 osteoblasts by CRISPR/dCas9-activation. **a** Overexpression as measured by relative mRNA levels at day 14 of osteoblastic differentiation. **b** TET2, KDM4C and KDM6A protein expression levels at day 14 of osteogenic differentiation as measured by western blot analysis. **c** 5hmC dot blot at day 14 of osteoblastic differentiation. Bar graphs represent mean ± SD, **p*<0.05; ***p*<0.01; ****p*<0.001. *N*=3, all from biological independent experiments and one-way ANOVA with Dunnett's multiple comparison test to NegCtrl (**a**). Source data as well as exact *p*=values for all comparisons in (**a**) are provided in the Source Data File.

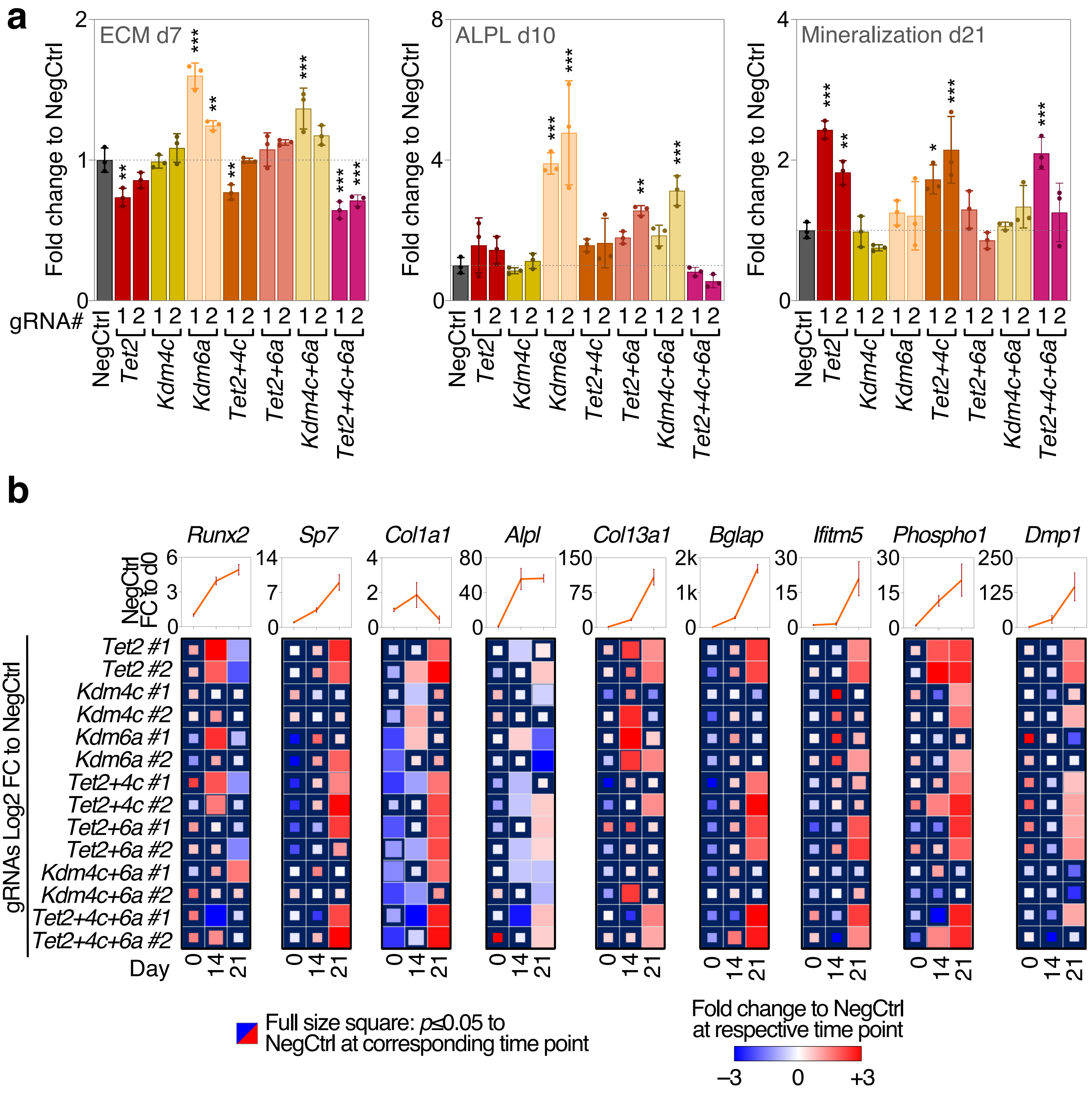

Supplementary Figure 16: Effects of combined TET2, KDM4C and KDM6A overexpression in differentiating MC3T3-E1 osteoblasts. **a** Relative ECM deposition, ALPL activity and ECM mineralization. **b** mRNA expression of osteogenic markers after overexpression of *Tet2*, *Kdm4c*, *Kdm6a* or combinations of these during osteogenic differentiation; line graphs on top represent mRNA expression for NegCtrls, heat maps underneath refer to fold changes (FC) to NegCtrls at respective time points. Bar graphs represent mean ± SD, **p*<0.05; ***p*<0.01; ****p*<0.001. *N*=3, all from biological independent experiments and one-way ANOVA with Dunnett's multiple comparison test to NegCtrl (**a**), two-way ANOVA with Dunnett's multiple comparison test to NegCtrl at corresponding time point (**b**). Source data as well as exact *p*=values for all comparisons in (**a**) are provided in the Source Data File.

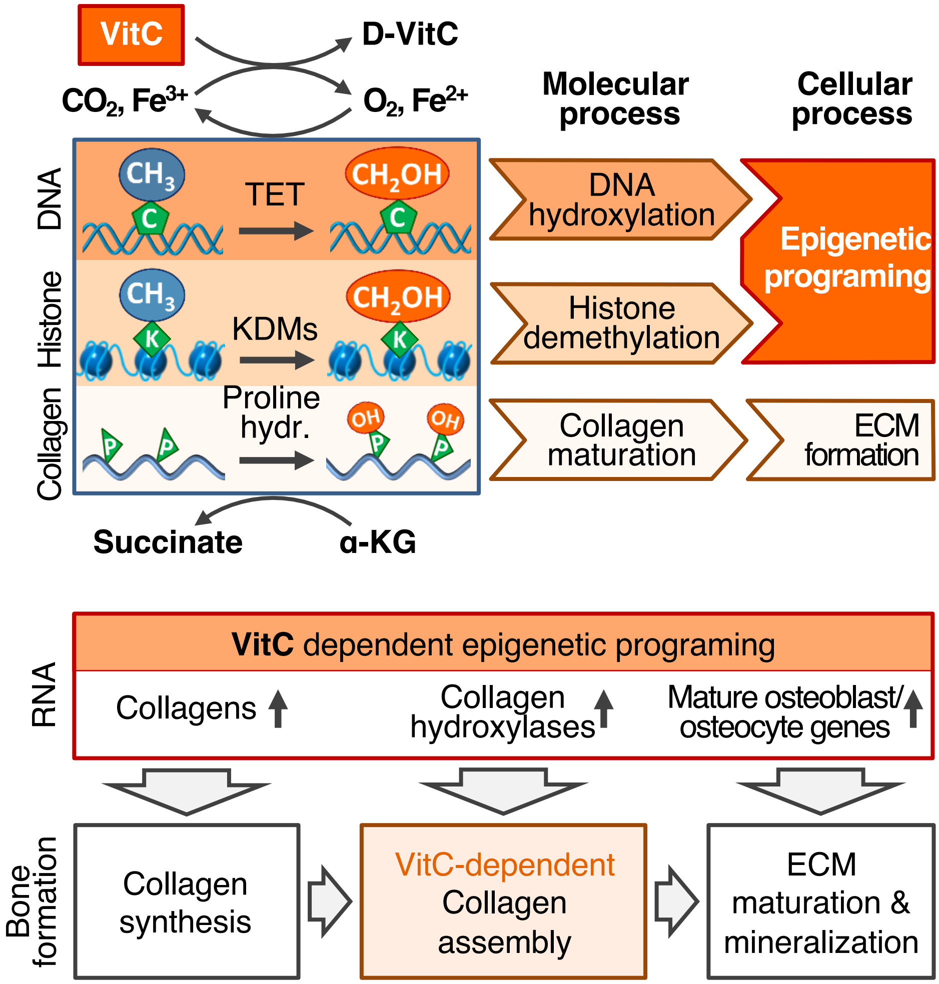

Supplementary Figure 17: Vitamin C orchestrates osteogenesis by enhancing the activity of DNA, histone and collagen hydroxylases. Vitamin C acts as co-factor for numerous hydroxylases with varying substrate specificities. During osteogenesis, Vitamin C epigenetically activates and sustains the expression of osteogenic genes, especially via hydroxylation of cytosines on DNA. These genes functionally address all osteoblastic features and functions and include osteogenic transcription factors, collagens, collagen hydroxylases and genes related to ECM mineralization. Source data are provided as a Source Data file.

Gene	Forward	Reverse
**Primers**		
Alpl	CCAGAAAGACACCTTGACTGTGG	TCTTGTCCGTGTCGCTCACCAT
Bglap2	GCAATAAGGTAGTGAACAGACTCC	CCATAGATGCGTTTGTAGGCGG
Bmp2	GGGACCCGCTGTCTTCTAGT	TCAACTCAAATTCGCTGAGGAC
Cebpa	GCAAAGCCAAGAAGTCGGTGGA	CCTTCTGTTGCGTCTCCACGTT
Cebpb	CAACCTGGAGACGCAGCACAAG	GCTTGAACAAGTTCCGCAGGGT
Col11a2	GAAGGGTGCTCGTGGGAAA	GAGGGCCTGGGTATCCTAGAG
Col1a1	CCTCAGGGTATTGCTGGACAAC	CAGAAGGACCTTGTTTGCCAGG
Dlx3	CACTGACCTGGGCTATTACAGC	GAGATTGAACTGGTGGTGGTAG
Dlx5	AGGCTTATGCCGACTACGGCTA	CTCTGGCTCCGCCACTTCTTTC
Dmp1	AAAGACCACGACAGTGAGGAT	CATCATCGAACTCAGAACCGTC
Enpp1	CTGGTTTTGTCAGTATGTGTGCT	CTCACCGCACCTGAATTTGTT
Grem1	CTGGGGACCCTACTGCCAA	TTTGCACCAATCTCGCTTCAG
Hprt1	CTGGTGAAAAGGACCTCTCGAAG	CCAGTTTCACTAATGACACAAACG
Ibsp	CAGGGAGGCAGTGACTCTTC	AGTGTGGAAAGTGTGGCGTT
Ifitm5	ACCACGAGATCACATGCTCTG	GGATGTTGTAGCACTTGGCTT
Igfbp5	CCCTGCGACGAGAAAGCTC	GCTCTTTTCGTTGAGGCAAACC
Jhdm1d	GGCAAACCAGTTAAATCTCAGGG	AGGTTAGAAGGAGTTCGGACAT
Kdm4a	CTATGGGCTCTGTGAGGCTG	GGATAAAAACCTGGAGCCTAAAGC
Kdm4b	TGACTCACAGGGAACAACCC	CTCCTGCTTGACCACAGACA
Kdm4c	CGAACAGCTGTCACCCAGT	GATGGCCACAGATGTTTCTGC
Kdm6a	TCAACATGCTCCTCCATTACCA	GCCAGCAGCCTTACGAGATA
Kdm6b	GTCAGCCTCATAGCAGGACC	CGCCTCAGTAACAGCCAGAT
Lox	TCTTCTGCTGCGTGACAACC	GAGAAACCAGCTTGGAACCAG
Loxl4	GCCAACGGACAGACCAGAG	CCAGGTCAAGGCTGACTCAAA
Lum	CTCTTGCCTTGGCATTAGTCG	GGGGGCAGTTACATTCTGGTG
Mef2c	GTGGTTTCCGTAGCAACTCCTAC	GGCAGTGTTGAAGCCAGACAGA
Mepe	AATGCCCAGAGACTAAGCCC	CTGTCTTCATTCGGCATTGGT
Mmp13	CTTCTTCTTGTTGAGCTGGACTC	CTGTGGAGGTCACTGTAGACT
Msx2	AAGACGGAGCACCGTGGATACA	CGGTTGGTCTTGTGTTTCCTCAG
Nrip2	CAGCAGCGCCAACTCAAAC	GGCGTCTTTGGATCACACTGT
P3h1	AACAGAAGTCGGAACGCGAAA	TCCACGAGGGTCTCGATCTC
P3h2	TGTGGCCGAATGATCAGCTT	GTATCCGCTCCAGTTCTCGG
P3h3	TGTCCTGAGTGTCCTGCTCT	CCGGGTCCTTGAAGCTAGTG
P4ha1	TGGGTTGTTTTAATGATGGCGA	GCCGGTCTAACTTCTCTGCC
P4ha2	CAGGTACTATGATGTGATGTCCG	AAAGGGTCGCCTTGAGAAGTC
P4ha3	ACATGCTCAACGTGAAGGG	TGCCAACTTGGAAGCAGTCAT
Phex	GAAAGGGGACCAACCGAGG	AACTTAGGAGACCTTGACTCACT
Phospho1	ATGAGCGGGTGTTTTCCAG	TGCCGTCCCTAGATAGGCATC
Plin1	CAAGCACCTCTGACAAGGTTC	GTTGGCGGCATATTCTGCTG
Plod1	GAAGGATGACGCCAAGCTAGA	TGAAGAACTGAGCTGAACGCT
Plod2	GAGAGGCGGTGATGGAATGAA	ACTCGGTAAACAAGATGACCAGA
Plod3	ATGTGGCTCGAACAGTTGGTG	TTGCCAGAATCACGTCGTAGC
Pparg	TCGCTGATGCACTGCCTATG	GAGAGGTCCACAGAGCTGATT
Runx2	CCTGAACTCTGCACCAAGTCCT	TCATCTGGCTCAGATAGGAGGG
Slc2a1	CAGTTCGGCTATAACACTGGTG	GCCCCCGACAGAGAAGATG
Slc2a3	ATGGGGACAACGAAGGTGAC	CAGGTGCATTGATGACTCCAG
Slc36a2	GCATAACCGGGTTCCAGACAT	ATGAGGCCCATTACCAGCAAG
Smpd3	ACACGACCCCCTTTCCTAATA	GGCGCTTCTCATAGGTGGTG
Sost	AGCCTTCAGGAATGATGCCAC	CTTTGGCGTCATAGGGATGGT
Sp7	GGCTTTTCTGCGGCAAGAGGTT	CGCTGATGTTTGCTCAAGTGGTC
Sparc	GTGGAAATGGGAGAATTTGAGGA	CTCACACACCTTGCCATGTTT
Spp1	GCTTGGCTTATGGACTGAGGTC	CCTTAGACTCACCGCTCTTCATG
Tet1	ATTTCCGCATCTGGGAACCTG	GGAAGTTGATCTTTGGGGCAAT
Tet2	CCCGTTAGCAGAGAGACCTCA	CTGACTGTGCGTTTTATCCCT
Tet3	CGCTGCTCGTCTGGAAGATG	GGCCCCGTAAGATGACACA
Wnt11	GCTGGCACTGTCCAAGACTC	CTCCCGTGTACCTCTCTCCA
Wnt5b	AGATAGGTAGCCGAGAGACTGC	GGTAGCCGTACTCCACGTTG
Wnt7b	TTTGGCGTCCTCTACGTGAAG	CCCCGATCACAATGATGGCA
Wnt9a	GGCCCAAGCACACTACAAG	AGAAGAGATGGCGTAGAGGAAA
Zfp521	GCGAAACCGAGATCCCTCAAA	GTCGCTCAGTGACTCAAACAC
**gRNAs**		
Kdm4a gRNA1	ACCGAACACTCACTACACCGACT	AAACAGTCGGTGTAGTGAGTGTTC
Kdm4a gRNA2	ACCGCAGTGGCCCCAGTCCCCGG	AAACCCGGGGACTGGGGCCACTGC
Kdm4b gRNA1	ACCGTGGGTGCCGGCACGTCCTC	AAACGAGGACGTGCCGGCACCCAC
Kdm4b gRNA2	ACCGGCCGCTCGGTGTTGATGAG	AAACCTCATCAACACCGAGCGGCC
Kdm4c gRNA1	ACCGGCTGGGCCGGGCGCTCTCT	AAACAGAGAGCGCCCGGCCCAGCC
Kdm4c gRNA2	ACCGGGGTCTCTAAACTGCTTAA	AAACTTAAGCAGTTTAGAGACCCC
Kdm6a gRNA1	ACCGGGCACGTCTGTCCGGAGTA	AAACTACTCCGGACAGACGTGCCC
Kdm6a gRNA2	ACCGGATCTGTGGCGGCCTCGGT	AAACACCGAGGCCGCCACAGATCC
Kdm6b gRNA1	ACCGCTCCTAAGGGAGATGATCG	AAACCGATCATCTCCCTTAGGAGC
Kdm6b gRNA2	ACCGCGAGTGCCGCTGCTCCTAA	AAACTTAGGAGCAGCGGCACTCGC
Jhdm1d gRNA1	ACCGCGGAGACCGAGAACGTGCG	AAACCGCACGTTCTCGGTCTCCGC
Jhdm1d gRNA2	ACCGGCGCGAGCGAGCAAGGTCG	AAACCGACCTTGCTCGCTCGCGCC
Tet2 gRNA1	ACCGGACGTGACTGGCATGCGAG	AAACCTCGCATGCCAGTCACGTCC
Tet2 gRNA2	ACCGCGGCACCCGGGGCTCAGGAA	AAACTTCCTGAGCCCCGGGTGCCGC

Supplementary Table 1: Sequences for used primers and gRNAs.

Mouse

Tissue	GEO annotation	SRR code
Bone	This study	This study
Bone	This study	This study
Bone	This study	This study
Bone	This study	This study
Bone	This study	This study
Bone	This study	This study
Immature cartilage	GSM2977013	SRR6666076
Immature cartilage	GSM2977014	SRR6666077
Immature cartilage	GSM2977015	SRR6666078
Mature cartilage	GSM2977016	SRR6666079
Mature cartilage	GSM2977017	SRR6666080
Mature cartilage	GSM2977018	SRR6666081
Cartilage hypertropic	GSM3154535	SRR7217931, SRR7217932, SRR7217933, SRR7217934, SRR7217935, SRR7217936
Cartilage hypertropic	GSM3154536	SRR7217937, SRR7217938, SRR7217939, SRR7217940, SRR7217941, SRR7217942
Cartilage hypertropic	GSM3154537	SRR7217943, SRR7217944, SRR7217945, SRR7217946, SRR7217947, SRR7217948
Cartilage hypertropic	GSM3154538	SRR7217949, SRR7217950, SRR7217951, SRR7217952, SRR7217953, SRR7217954
Cartilage hypertropic	GSM3154539	SRR7217955, SRR7217956, SRR7217957, SRR7217958, SRR7217959, SRR7217960
Cartilage prolif. zone	GSM3154555	SRR7218051, SRR7218052, SRR7218053, SRR7218054, SRR7218055, SRR7218056
Cartilage prolif. zone	GSM3154556	SRR7218057, SRR7218058, SRR7218059, SRR7218060, SRR7218061, SRR7218062
Cartilage prolif. zone	GSM3154557	SRR7218063, SRR7218064, SRR7218065, SRR7218066, SRR7218067, SRR7218068
Cartilage prolif. zone	GSM3154558	SRR7218069, SRR7218070, SRR7218071, SRR7218072, SRR7218073, SRR7218074
Cartilage prolif. zone	GSM3154559	SRR7218075, SRR7218076, SRR7218077, SRR7218078, SRR7218079, SRR7218080
Fat	GSM3093058	SRR6984624
Fat	GSM3093059	SRR6984625
Fat	GSM3093060	SRR6984626
Fat	GSM3093046	SRR6984612
Fat	GSM3093047	SRR6984613
Fat	GSM3093048	SRR6984614
Heart	GSM2595506	SRR5494677
Heart	GSM2595507	SRR5494678
Heart	GSM2595508	SRR5494679
Heart	GSM2595509	SRR5494680
Liver	GSM2428116	SRR5106551
Liver	GSM2428117	SRR5106553
Liver	GSM2428118	SRR5106555
Liver	GSM2452248	SRR5164499
Liver	GSM2452250	SRR5164501
Muscle	GSM2208774	SRR3705519
Muscle	GSM2208775	SRR3705520
Muscle	GSM2208779	SRR3705524
Muscle	GSM2208782	SRR3705527
Muscle	GSM2208783	SRR3705528
Spinal cord	GSM2836602	SRR6237215
Spinal cord	GSM2836603	SRR6237216
Spinal cord	GSM2836604	SRR6237217
Spinal cord	GSM2836605	SRR6237218
Spinal cord	GSM2836606	SRR6237219
Lung	GSM2927614	SRR6456618
Lung	GSM2927615	SRR6456619
Lung	GSM2927600	SRR6456604
Lung	GSM2927601	SRR6456605
Lung	GSM2927602	SRR6456606
Brain	GSM3169030	SRR7244449
Brain	GSM3169031	SRR7244450
Brain	GSM3169032	SRR7244451
Whole blood	GSM3244452	SRR7472435
Whole blood	GSM3244453	SRR7472436
Whole blood	GSM3244454	SRR7472437
Whole blood	GSM3244455	SRR7472438
BMSCs	GSM3155166	SRR7219450
BMSCs	GSM3155167	SRR7219451
BMSCs	GSM3242777	SRR7465595
BMSCs	GSM3242778	SRR7465596
BMSCs	GSM3242779	SRR7465597
BMSCs	GSM2793815	SRR6076938
BMSCs	GSM4238081	SRR10801329
BMSCs	GSM4238082	SRR10801330
BMSCs	GSM4238083	SRR10801331
ESCs	GSM4104491	SRR10212910
ESCs	GSM4104492	SRR10212911
ESCs	GSM5048817	SRR13572970
ESCs	GSM5048818	SRR13572971
ESCs	GSM5048819	SRR13572972
ESCs	GSM4850905	SRR12884018
ESCs	GSM4850906	SRR12884019
ESCs	GSM4850907	SRR12884020
ESCs	GSM4850908	SRR12884021
BMSCs (H3K27ac ChIP-Seq)	GSM1973729	SRR3020952
BMSCs (H3K27ac ChIP-Seq)	GSM1973730	SRR3020953

Human

Tissue	GEO annotation	SRR code
Bone	GSM1872881	SRR2305536
Bone	GSM1872882	SRR2305537
Bone	GSM1872883	SRR2305538
Bone	GSM1872884	SRR2305539
Bone	GSM1872885	SRR2305540
Bone	GSM2871626	SRR6337317
Bone	GSM2871627	SRR6337318
Bone	GSM2871628	SRR6337319
Adult cartilage	GSM2871645	SRR6337336
Adult cartilage	GSM2871646	SRR6337337
Adult cartilage	GSM2871647	SRR6337338
Juvenile cartilage	GSM2871642	SRR6337333
Juvenile cartilage	GSM2871643	SRR6337334
Juvenile cartilage	GSM2871644	SRR6337335
Tendon	GSM2871632	SRR6337323
Tendon	GSM2871633	SRR6337324
Tendon	GSM2871634	SRR6337325
White fat	GSM3119101	SRR7073362
White fat	GSM3119103	SRR7073364
White fat	GSM3119107	SRR7073368
White fat	GSM3119109	SRR7073370
White fat	GSM3119112	SRR7073373
Ligament	GSM2871629	SRR6337320
Ligament	GSM2871630	SRR6337321
Ligament	GSM2871631	SRR6337322
Brain	GSM1414931	SRR1424658
Brain	GSM1414947	SRR1424674
Brain	GSM1414962	SRR1424689
Brain	GSM1414952	SRR1424679
Liver	GSM2809210	SRR6163894
Liver	GSM2809211	SRR6163895
Liver	GSM2809213	SRR6163897
Liver	GSM2809214	SRR6163898
Muscle	GSM2835494	SRR6231603, SRR6231604, SRR6231605, SRR6231606
Muscle	GSM2835495	SRR6231607, SRR6231608, SRR6231609, SRR6231610
Muscle	GSM2835496	SRR6231611, SRR6231612, SRR6231613, SRR6231614
Muscle	GSM2835497	SRR6231615, SRR6231616, SRR6231617, SRR6231618
Lung	GSM1194676	SRR942541
Lung	GSM1194682	SRR942547
Lung	GSM1194687	SRR942552
Lung	GSM1194693	SRR942558
Whole blood	GSM3056569	SRR6868715
Whole blood	GSM3056570	SRR6868716
Whole blood	GSM3056571	SRR6868717
Whole blood	GSM3056572	SRR6868718
Whole blood	GSM3056573	SRR6868719
Whole blood	GSM3056574	SRR6868720

Supplementary Table 2: Accession codes for publically available RNA-Seq datasets used in this study.

We note that ▭ marks picture shown in corresponding figure.

Uncropped blots: Supplementary Figure 3c

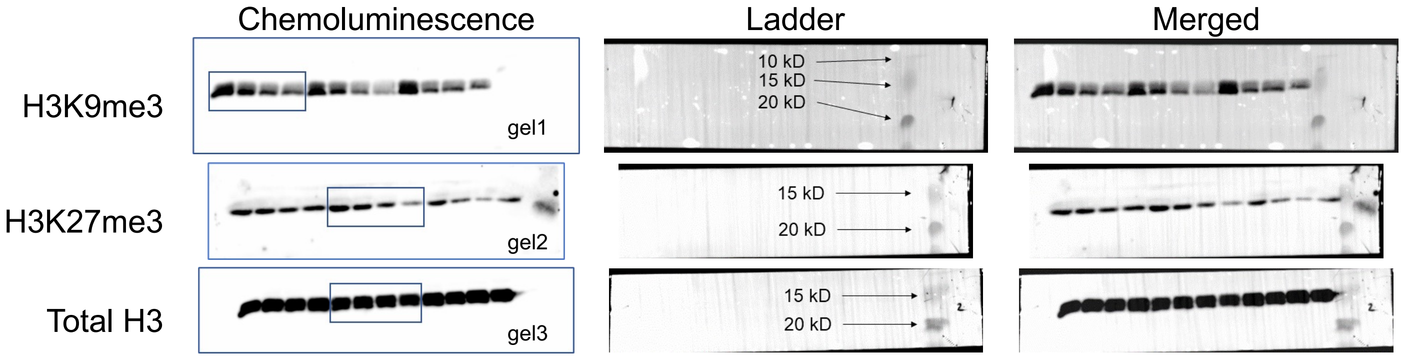

Uncropped blots: Supplementary Figure 4e

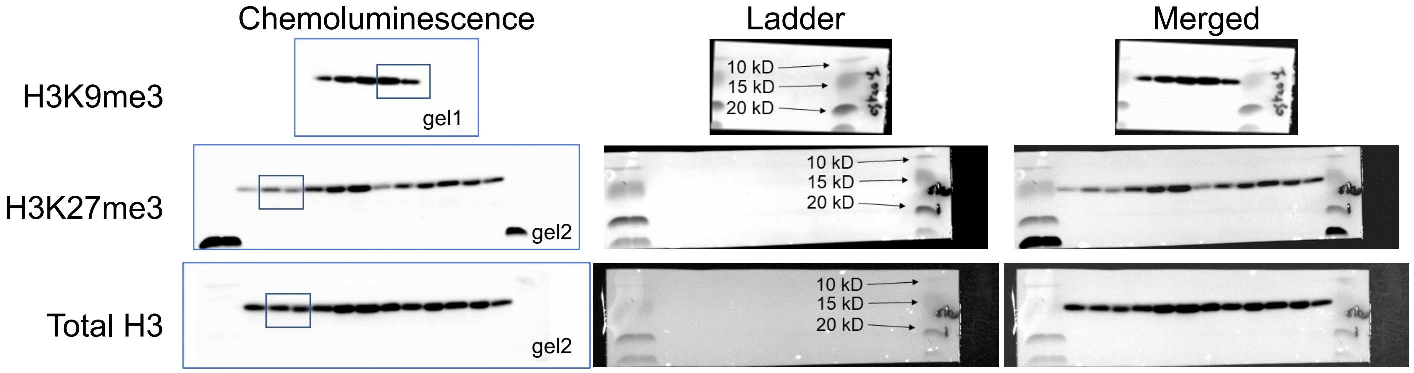

Uncropped blots: Supplementary Figure 5d

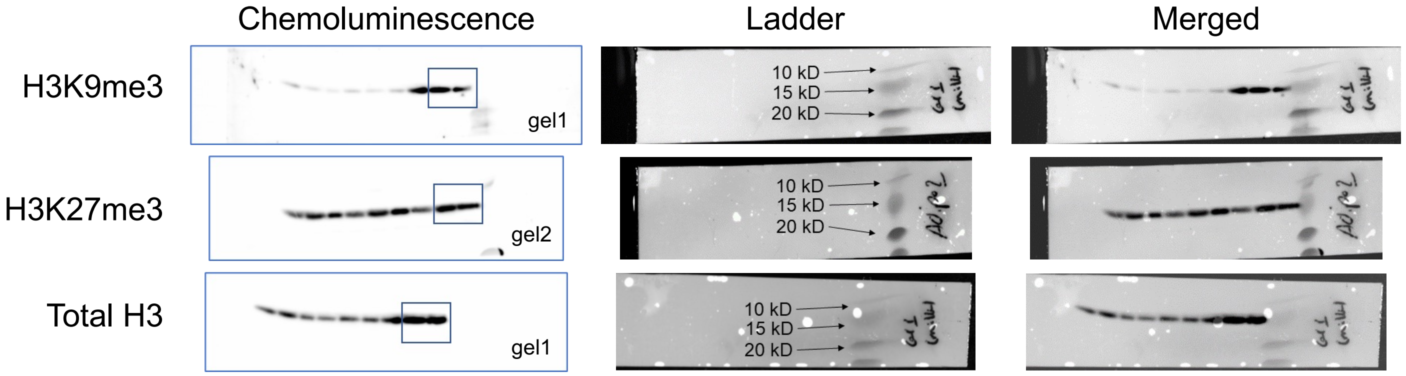

Uncropped blots: Supplementary Figure 6b

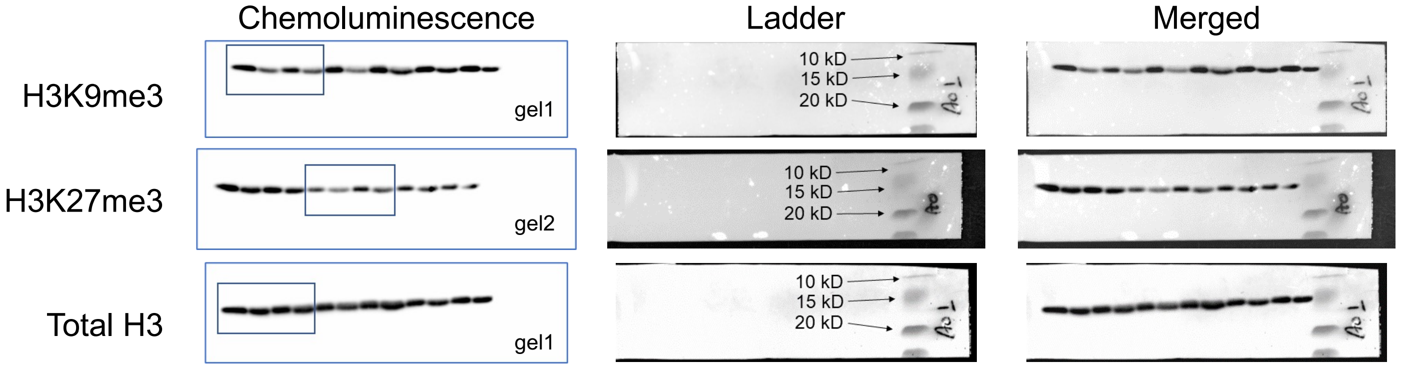

We note that ▭ marks picture shown in corresponding figure.

Uncropped blots: Supplementary Figure 8c

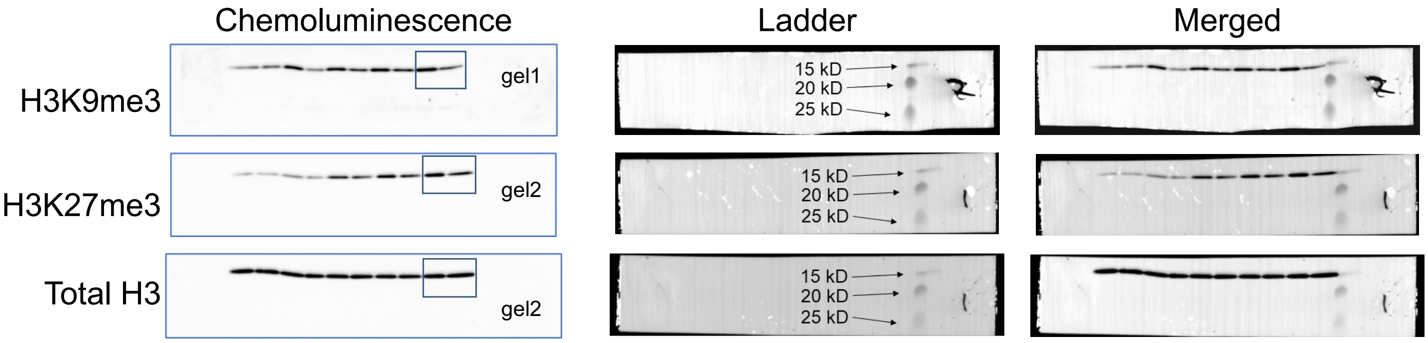

Uncropped blots: Supplementary Figure 15b

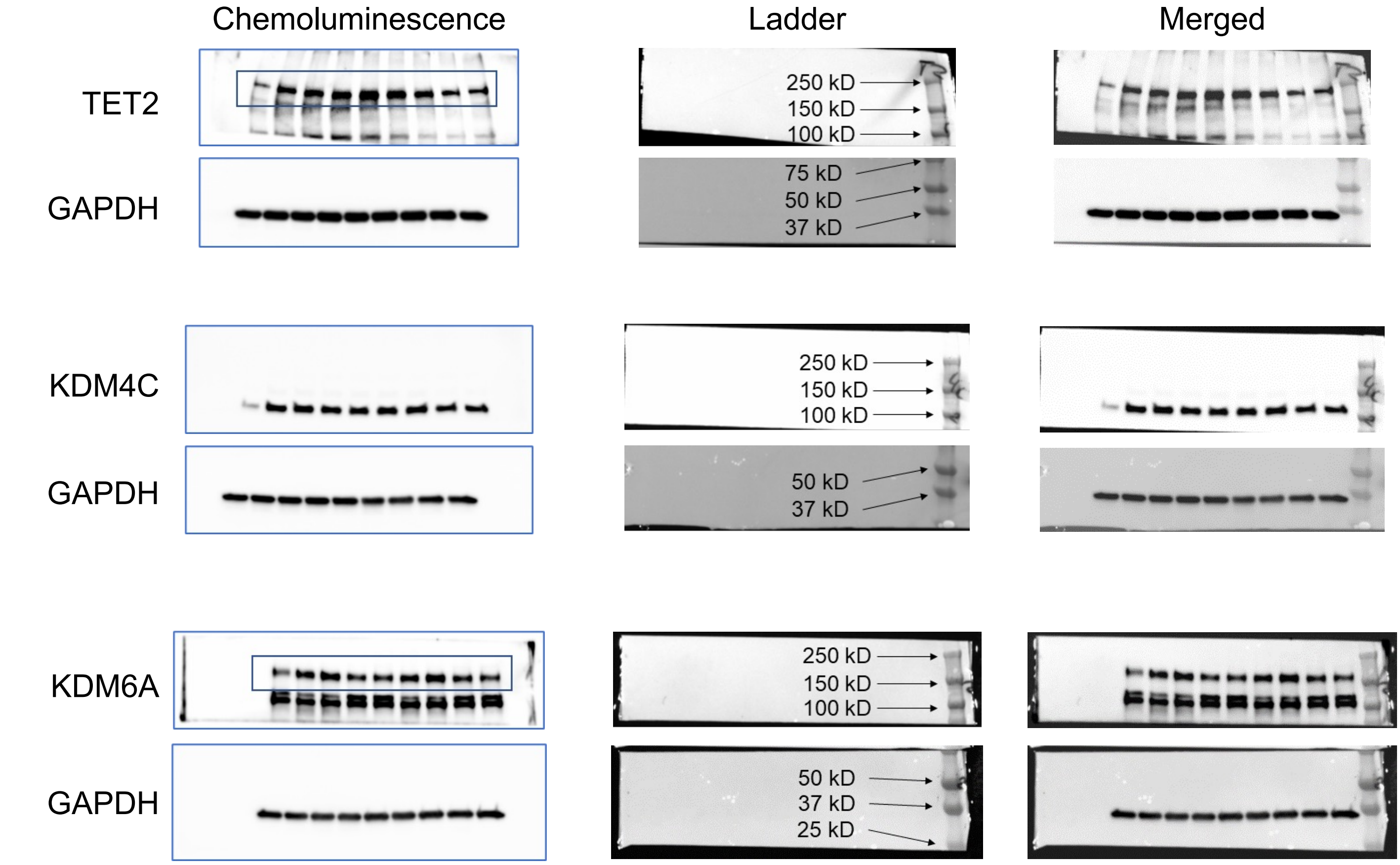

Supplement: Supplementary file 1 — Supplementary Information [file 41467_2022_32915_MOESM1_ESM.pdf]
