# Supplementary material for: Vitamin C epigenetically controls osteogenesis and bone mineralization
Source: Nat Commun. 2022 Oct 6;13:5883. doi: 10.1038/s41467-022-32915-8 (PMC9537512; doi:10.1038/s41467-022-32915-8)
Supplement: Supplementary file 3 — Reporting Summary [file 41467_2022_32915_MOESM3_ESM.pdf]

nature portfolio

Corresponding author(s): Roman Thaler, Andre van Wijnen

Last updated by author(s): Jul 6, 2022

Reporting Summary

Nature Portfolio wishes to improve the reproducibility of the work that we publish. This form provides structure for consistency and transparency in reporting. For further information on Nature Portfolio policies, see our Editorial Policies and the Editorial Policy Checklist.

Statistics

For all statistical analyses, confirm that the following items are present in the figure legend, table legend, main text, or Methods section.

n/a	Confirmed	
☐	☒	The exact sample size (*n*) for each experimental group/condition, given as a discrete number and unit of measurement
☐	☒	A statement on whether measurements were taken from distinct samples or whether the same sample was measured repeatedly
☐	☒	The statistical test(s) used AND whether they are one- or two-sided<br>*Only common tests should be described solely by name; describe more complex techniques in the Methods section.*
☒	☐	A description of all covariates tested
☐	☒	A description of any assumptions or corrections, such as tests of normality and adjustment for multiple comparisons
☐	☒	A full description of the statistical parameters including central tendency (e.g. means) or other basic estimates (e.g. regression coefficient) AND variation (e.g. standard deviation) or associated estimates of uncertainty (e.g. confidence intervals)
☐	☒	For null hypothesis testing, the test statistic (e.g. *F*, *t*, *r*) with confidence intervals, effect sizes, degrees of freedom and *P* value noted<br>*Give P values as exact values whenever suitable.*
☒	☐	For Bayesian analysis, information on the choice of priors and Markov chain Monte Carlo settings
☒	☐	For hierarchical and complex designs, identification of the appropriate level for tests and full reporting of outcomes
☒	☐	Estimates of effect sizes (e.g. Cohen's *d*, Pearson's *r*), indicating how they were calculated

Our web collection on statistics for biologists contains articles on many of the points above.

Software and code

Policy information about availability of computer code

Data collection	The Biorad CFX384 Real Time System was used for rt-qPCR measurements.<br>The Zeiss Axio Vert.A1 and Zeiss LSM 780 microscopes were used for bright-field and confocal cell and immunofluorescence imaging.<br>A Tecan Infinite F200 Pro multiwell plate reader was used for cell-culture based measurements involving fluorescence and absorbance.<br>RNA-Seq, ChIP-Seq and MeDIP-Seq were performed on an Illumina HiSeq 4000 instrument in the Mayo Clinic Genome Facility Sequencing Core.<br>The Biorad Chemidoc Touch Imaging System was used for western blot and dot blot image acquisition.<br>The Epson Perfection V700 Photo scanner was used to obtain scans of cell culture plates with mineralizing cell cultures as well a colony forming cell units.<br>Bone density data was obtained by microcomputed tomography on a VivaCT 40 scanner, SCANCO Medical AG.<br>Tree point bending testing was performed on a Model 312 device, MTS Systems.<br>Spectra for the measurement of collagen cross-linking ratios were obtained with a Bruker Equinox 55 spectrometer coupled to a Bruker Hyperion 3000 FTIR microscope.
Data analysis	rt-qPCR data was analyzed with the Biorad CFX Manager 3.0, 2012 software.<br>Bright-field, fluorescent and confocal microscopy images were analyzed using Zen blue edition software version 2.3 and Zen black edition software version 14.0 as well as ImageJ software (National Institute of Health, Bethesda, MD).<br>Magellan software version 7.2 was used for the acquisition and analysis of all data generated by the Tecan Infinite F200 Pro multiwell plate reader.<br>Western blot and dot blot image analysis and quantification was performed using the Biorad Image Lab software, version 5.2.1.<br>Reconstruction, analysis and visualization of microcomputed tomography data was performed with the SCANCO Reconstruction software.

Raw reads from RNA-seq, ChIP-seq of MeDIP-Seq experiments were assessed for run quality using fastqc version 0.72. Alignment of RNA-Seq raw reads to reference genome was performed using RNA STAR Galaxy version 2.6.0b-1 software. Differential gene expression analysis was performed using featureCounts Galaxy version 1.6.3 and DESeq2 Galaxy version 2.11.40.2. Fragments per kilobase of exon per million mapped fragments (FPKM) was performed by cufflinks v2.2.0. For normalization of possible batch effects between runs, RUVSeq (Galaxy Version 1.26.0) was applied. Gene set enrichment analysis was performed with GSEA version 4.0.1 (Broad Institute).
Mapping of MeDIP-Seq or ChIPSeq data to reference genome was performed using BWA Galaxy version 0.7.15.1. Peak calling was performed with MACS2 callpeak Galaxy version 2.1.0.20140616.0 and differential peak analysis with DiffBind Galaxy version 2.6.6.4 or 2.10.0. Peak size and location was visualized with IGV version 2.3.92.MAYO. Genomic 5hmC and ChIP-Seq related peak distribution was assessed with CEAS software (Version1.0.0, Cistrome, Liu Lab). Super-enhancer assessment was performed by using the package the software NaviSE (Ascension M e al., BMC Bioinformatics 2017). Overlapping SE between samples and/or treatments were assessed with Bedtools (version 2.29). MeDIP-Seq and ChIP-Seq based gene-ontology was performed using GREAT version 3.0.0 (Bejerano Lab, Stanford; http://great.stanford.edu/public/html/).
Hierarchical clustering and heat map generation was executed with Morpheus (Broad Institute, https://software.broadinstitute.org/morpheus/).
Statistical data analysis and figure generation was performed using GraphPad Prism software, version 8.

For manuscripts utilizing custom algorithms or software that are central to the research but not yet described in published literature, software must be made available to editors and reviewers. We strongly encourage code deposition in a community repository (e.g. GitHub). See the Nature Portfolio guidelines for submitting code & software for further information.

Data

Policy information about availability of data

All manuscripts must include a data availability statement. This statement should provide the following information, where applicable:

- Accession codes, unique identifiers, or web links for publicly available datasets
- A description of any restrictions on data availability
- For clinical datasets or third party data, please ensure that the statement adheres to our policy

All data needed to evaluate the conclusions of the study are present in the paper and/or the Supplementary Materials. Raw and processed NGS data are deposited in the National Center for Biotechnology Information GEO database under accession number GSE138854.

Human research participants

Policy information about studies involving human research participants and Sex and Gender in Research.

Reporting on sex and gender

Use the terms sex (biological attribute) and gender (shaped by social and cultural circumstances) carefully in order to avoid confusing both terms. Indicate if findings apply to only one sex or gender; describe whether sex and gender were considered in study design whether sex and/or gender was determined based on self-reporting or assigned and methods used. Provide in the source data disaggregated sex and gender data where this information has been collected, and consent has been obtained for sharing of individual-level data; provide overall numbers in this Reporting Summary. Please state if this information has not been collected. Report sex- and gender-based analyses where performed, justify reasons for lack of sex- and gender-based analysis.

Population characteristics

Describe the covariate-relevant population characteristics of the human research participants (e.g. age, genotypic information, past and current diagnosis and treatment categories). If you filled out the behavioural & social sciences study design questions and have nothing to add here, write "See above."

Recruitment

Describe how participants were recruited. Outline any potential self-selection bias or other biases that may be present and how these are likely to impact results.

Ethics oversight

Identify the organization(s) that approved the study protocol.

Note that full information on the approval of the study protocol must also be provided in the manuscript.

Field-specific reporting

Please select the one below that is the best fit for your research. If you are not sure, read the appropriate sections before making your selection.

☒ Life sciences ☐ Behavioural & social sciences ☐ Ecological, evolutionary & environmental sciences

For a reference copy of the document with all sections, see nature.com/documents/nr-reporting-summary-flat.pdf

Life sciences study design

All studies must disclose on these points even when the disclosure is negative.

Sample size

Animal experiments for the assessment of structural and physical bone parameters, sample sizes for individual experimental groups were determined by power statistics calculations based on a co-efficient of variation of 25% in the types of data that were collected and after accepting a 5% error probability. For in vitro experiments, sample sizes were based on previously published experiments where differences

were observed:
- Paradise C. et al., 2022 (PMID: 34700039), Brd4 is required for chondrocyte differentiation and endochondral ossification
- Khani F. et al., 2017 (PMID: 27862226), Histone H4 Methyltransferase Suv420h2 Maintains Fidelity of Osteoblast Differentiation
- Thaler R. et al., 2016 (PMID: 26757819), Anabolic and Antiresorptive Modulation of Bone Homeostasis by the Epigenetic Modulator Sulforaphane, a Naturally Occurring Isothiocyanate
- Dudakovic A. et al., 2015 (PMID: 26424790), Epigenetic Control of Skeletal Development by the Histone Methyltransferase Ezh2
- Thaler R. et al., 2015 (PMID: 25491310), Acute-phase protein serum amyloid A3 is a novel paracrine coupling factor that controls bone homeostasis

Data exclusions

No samples were excluded from analysis.

Replication

The exact number of replicates in groups or independent biological experiments is mentioned in the figures or figure legends. For all results presented, attempts at replication were successful.

Randomization

For experiments involving vitamin C deprivation in mice, animals were randomly allocated in the treatment groups. For microscopy image capturing and analysis, selected areas were chosen randomly. For other experiments, randomization was not applicable and/or relevant as these experiments did not involve clinical trials or population based studies. However, for the in vitro performed experiments, cells were cultured under the described conditions and unbiasedly seeded to well positions and treatments. In addition, cell harvesting, processing and analysis was performed in random order.

Blinding

Tissue and cell culture collections for molecular analysis were not performed blind as subsequent experiments were carried out by the same investigator and knowledge of sample identity were necessary for proper data collection and evaluation. Furthermore, for experiments involving microscopy imaging investigators were not blinded to treatment groups as knowledge of experimental procedures, conditions and groups were necessary for proper data collection and evaluation. Data generation and collection from Mayo core facilities (micro CT data collection, tri-point bending tests as well as ChiP and MeDIP-Seq) was performed blind as the handling investigators were not aware of the sample identity during data collection/generation.

Reporting for specific materials, systems and methods

We require information from authors about some types of materials, experimental systems and methods used in many studies. Here, indicate whether each material, system or method listed is relevant to your study. If you are not sure if a list item applies to your research, read the appropriate section before selecting a response.

Materials & experimental systems

n/a	Involved in the study
☐	☒ Antibodies
☐	☒ Eukaryotic cell lines
☒	☐ Palaeontology and archaeology
☐	☒ Animals and other organisms
☒	☐ Clinical data
☒	☐ Dual use research of concern

Methods

n/a	Involved in the study
☐	☒ ChIP-seq
☒	☐ Flow cytometry
☒	☐ MRI-based neuroimaging

Antibodies

Antibodies used

5hmC antibody, Active Motif, 39769, 1:5,000 (IF);
5hmC antibody, Active Motif, 39791, 0.2 µg/ml (DB), 1µg antibody/µg DNA (MeDIP-Seq);
5mC, Diagenode, C15200081, 33D3; 1:300 (DB);
5fC, Millipore, MABE1092, FC-5; 0.5 µg/ml (DB);
H3K9me3, Diagenode, C15410056, A2810P, 1 µg/IP (ChIP-Seq);
H3K9me3, Active Motif, 39062, 1:3000 (WB);
H3K27me3, Cell Signaling Technology, 9733BC, C36B11, 1:1000 (WB), 1:50 (ChIP-Seq);
Total histone 3, Millipore-Sigma, 06-755, 1:5000 (WB);
KDM4C, Novus Biologicals, NBP1-49600, 1:10000 (WB);
KDM6A, Novus biologicals, NBP1-80628, 0.1 µg/ml (WB);
TET1, Genetex, GTX124207, N3C1, 1:1000, (WB);
TET2, Abcam, ab124297, 1:250, (WB);
BGLAP, Abcam, ab13421, 10 µg/ml (IF);
GAPDH, Cell signaling Technology, 5174, 1:1000 (WB);
Goat anti-rabbit IgG Alexa-647 -conjugated secondary antibody, Thermo Fisher, A-21245, 1:400 (IF);
Goat anti-mouse IgG Alexa-488 -conjugated secondary antibody, Thermo Fisher, A-11001; 1:400 (IF);
anti rabbit IgG, HRP-conjugated secondary antibody, Cell signaling Technology, 7074, 1:5000 (WB);
anti mouse IgG, HRP-conjugated secondary antibody, Cell signaling Technology, 7076; 1:5000 (WB);
normal rabbit IgG, Santa Cruz, #sc-2027, 1:100 (IF).

Validation

All antibodies used in this study are commercially available and all are validated by the vendors for the specific assays and species used; the validation data is available on the vendors website. As stated below, some antibodies are additionally validated in our study

as well.
5hmC antibody, Active Motif, 39769; https://www.activemotif.com/catalog/details/39769/5-hydroxymethylcytidine-5-hmc-antibody. The manufacturer has validated this antibody for MeDIP and DB in the species human and mouse. Published applications for this antibody further include MeDIP-Seq, ICC/IF, IHC, FC and ELISA as in PMID: 27207465, PMID: 29130343, PMID: 27164004 and others.
5hmC antibody, Active Motif, 39791; https://www.activemotif.com/catalog/details/39791/5-hydroxymethylcytidine-antibody-pab. The manufacturer has validated this antibody for MeDIP, IHC and DB in the species human and mouse. Published applications for this antibody further include MeDIP-Seq and ICC/IF as in PMID: 30700309, PMID: 27489048 and others.
5mC, Diagenode, C15200081, 33D3; https://www.diagenode.com/en/p/5-mc-monoclonal-antibody-33d3-premium-100-ug-50-ul#. The manufacturer has validated this antibody for MeDIP, MeDIP-Seq, DB and IF in the species human, mouse, rat, cow, and others. Publications referring to this antibody include PMID: 31432508, PMID: 28232479, PMID: 29472622 and others.
5fC, Millipore, MABE1092, FC-5; https://www.emdmillipore.com/US/en/product/Anti-5-Formylcytosine-5fC-Antibody-clone-EDL-FC-5,MM_NF-MABE1092. The manufacturer has validated this antibody for DB and MeDIP. The antibody is not species specific.
H3K9me3 (ChiP-Seq), Diagenode, C15410056, A2810P; https://www.diagenode.com/en/p/h3k9me3-polyclonal-antibody-classic-50-ug. The manufacturer has validated this antibody for ChIP, ChIP-Seq, ELISA, DB and WB for the species human, mouse, zebrafish, trout, Daphnia, Arabidopsis and Drosophila. Publications referring to this antibody include PMID: 31053162, PMID: 25488809, PMID: 29478809 and others.
H3K9me3, Active Motif, 39062; https://www.activemotif.com/catalog/details/39161/histone-h3-trimethyl-lys9-antibody-pab. The manufacturer has validated this antibody for ChIP, ChIP-Seq, ICC/IF, WB and CUT and Tag in the species fission yeast, human and predicted to work in a wide range of other organisms. Published applications referring to this antibody are found in PMID: 34386729, PMID: 35110421 and others.
H3K27me3, Cell Signaling Technology, 9733BC, C36B11; https://www.cellsignal.com/products/primary-antibodies/tri-methyl-histone-h3-lys27-c36b11-rabbit-mab/9733?site-search-type=Products&N=4294956287&Ntt=9733bc&fromPage=plp&_requestid=2892850. The manufacturer has validated this antibody for ChIP, ChIP-Seq, IHC, IF, Cut&Run and WB in the species human, mouse, rat and Monkey. Published applications referring to this antibody are found in PMID: 35399730, PMID: 35093191, PMID: 33688077 and others.
Total Histone 3, Millipore-Sigma, 06-755; https://www.emdmillipore.com/US/en/product/Anti-Histone-H3-Antibody,MM_NF-06-755. The manufacturer has validated this antibody for WB, ICC, IP and ChIP in the species human, rat and mouse.
KDM4C, Novus Biologicals, NBP1-49600; https://www.novusbio.com/products/lysine-k-specific-demethylase-4c-kdm4c-jmjd2c-antibody_nbp1-49600. In this manuscript validation for this antibody is found in suppl. figure 15b where compared to NegCtrls, overexpression of KDM4C in osteoblasts increases KDM4C signal in the related WB. Furthermore, the manufacturer has validated this antibody for WB, ChIP, ICC, IF and IHC in the species human and mouse. Published applications referring to this antibody are found in PMID: 30683841, PMID: 26397136 and others.
KDM6A, Novus biologicals, NBP1-80628; https://www.novusbio.com/products/kdm6a-antibody_nbp1-80628. In this manuscript validation for this antibody is found in suppl. figure 15b where compared to NegCtrls, overexpression of KDM6C in osteoblasts increases KDM6C signal in the related WB. Furthermore, the manufacturer has validated this antibody for WB, ICC, IF and IHC in the species human, mouse and rat. Published applications referring to this antibody are found in PMID 29029452, PMID 32427586 and others.
TET1, Genetex, GTX124207, N3C1; https://www.genetex.com/Product/Detail/TET1-antibody-N3C1/GTX124207. In this manuscript validation for this antibody is found in figure 10b where compared to Ctrls, in TET1 negative bone tissue TET1 cannot be detected by WB. Furthermore, the manufacturer has validated this antibody for WB, ICC, IF, IHC and ChIP in the species human, mouse and monkey. Published applications referring to this antibody are found in PMID: 34844636, PMID: 34731622 and others.
TET2, Abcam, ab124297; https://www.abcam.com/tet2-antibody-ab124297.html. In this manuscript validation for this antibody is found in figure 10b where compared to Ctrls, in TET2 negative bone tissues TET2 cannot be detected by WB and in figure 8a and suppl. figure 15b where compared to NegCtrls, overexpression of TET2 in osteoblasts increases TET2 signal in the related WB. Furthermore, the manufacturer has validated this antibody for ICC and WB in mouse samples. Published applications referring to this antibody are found in PMID: 34009618, PMID: 32444652, PMID: 31310587 and others.
BGLAP, Abcam, ab13421; https://www.abcam.com/osteocalcin-antibody-ocg4-ab13421.html > Download archived datasheet (PDF). The manufacturer has validated this antibody for : ELISA, WB, IHC, ICC, IF in mouse samples.
GAPDH, Cell signaling Technology, 5174; https://www.cellsignal.com/products/primary-antibodies/gapdh-d16h11-xp-rabbit-mab/5174. The manufacturer has validated this antibody for WB, IHC and IF in the species human, mouse, rat and monkey. Published applications referring to this antibody are found in PMID: 35093191, PMID: 34472491, PMID: 35265680 and others.
Goat anti-rabbit IgG Alexa-647 -conjugated secondary antibody, Thermo Fisher, A-21245; https://www.thermofisher.com/antibody/product/Goat-anti-Rabbit-IgG-H-L-Highly-Cross-Adsorbed-Secondary-Antibody-Polyclonal/A-21245. The manufacturer has validated this antibody for IF against rabbit IgG.
Goat anti-mouse IgG Alexa-488 -conjugated secondary antibody, Thermo Fisher, A-11001; https://www.thermofisher.com/antibody/product/Goat-anti-Mouse-IgG-H-L-Cross-Adsorbed-Secondary-Antibody-Polyclonal/A-11001. The manufacturer has validated this antibody for IF against mouse IgG.
Anti-rabbit IgG, HRP-conjugated secondary antibody, Cell signaling Technology, 7074; https://www.cellsignal.com/products/secondary-antibodies/anti-rabbit-igg-hrp-linked-antibody/7074. The manufacturer has validated this antibody for IF against rabbit IgG. Published applications referring to this antibody are found in PMID: 35401801, PMID: 35340435 and others.
Anti-mouse IgG, HRP-conjugated secondary antibody, Cell signaling Technology, 7076; https://www.cellsignal.com/products/secondary-antibodies/anti-mouse-igg-hrp-linked-antibody/7076. The manufacturer has validated this antibody for IF against mouse IgG. Published applications referring to this antibody are found in PMID: 35187508, PMID: 35244193 and others.

Eukaryotic cell lines

Policy information about cell lines and Sex and Gender in Research

Cell line source(s)	Gulo-/- mBMSCs and Dmp1-EGFPTopaz/Gulo-/- mBMSCs BMSCs; mice were sacrificed, and bone marrow aspirates were used to obtain cell cultures as described before (PMID: 19131962); both sexes were used.<br>hBMSCs, multiple donors, Lonza, PT-2501; aliquots from passage 3 were used for the experiments.<br>MC3T3-E1 cell-line, ATCC CRL-2593, subclone 4, aliquots from passages 4-10 were used for the experiments.

	MLO-A5 cells, gift from Dr. Lynda Bonewald, Indiana University School of Medicine, aliquots from passages 5-7 were used for the experiments.<br>HEK293 cells, ATCC CRL-1573, aliquots from passages 3-6 were used for the experiments.
Authentication	The Dmp1-EGFPTopaz/Gulo-/- mBMSCs were newly generated for this study. PCR-based genotyping was used for cell identity authentication. Furthermore, Dmp1-EGFP expression measured by a plate reader and visualized by confocal microscopy as well as the osteogenic mineralization and osteogenic gene expression profiles at advanced stages of osteogenic differentiation experiments confirmed cell identity. All other cell-lines used in this study are established cell lines which have been already authenticated by the vendors (MC3T3-E1, hBMSCs, HEK293) or the institutional supplier (MLO-A5) or were obtained with published and well known protocols designed to specifically obtain the used cell-line (mBMSCs; protocol published in PMID: 19131962). Therefore, this set of cell-lines were not authenticated once again in this study.
Mycoplasma contamination	All cells and cell lines were tested negative for mycoplasma.
Commonly misidentified lines<br>(See ICLAC register)	No commonly misidentified cell lines were used.

Animals and other research organisms

Policy information about studies involving animals; ARRIVE guidelines recommended for reporting animal research, and Sex and Gender in Research

Laboratory animals	Male and female mice were on the C57BL/6 genetic background and tissue and cell collection was performed at 11 and 20 weeks of age. Primary bone marrow stromal cell collection was performed as published before (PMID: 19131962). All mice were housed in a selected pathogen-free barrier environment with ad libitum access to food and water, 12-hour light and dark cycles, temperature between 20-26C and humidity at ~70%.
Wild animals	The study did not involve wild animals.
Reporting on sex	Findings reported in this study apply to both sexes as study design and experimental setups included both, male and female animals.
Field-collected samples	The study did not involve samples collected from the field.
Ethics oversight	Experiments involving mice were reviewed and approved by the Mayo Clinic Institutional Animal Care and Use Committee.

Note that full information on the approval of the study protocol must also be provided in the manuscript.

ChIP-seq

Data deposition

☒ Confirm that both raw and final processed data have been deposited in a public database such as GEO.

☒ Confirm that you have deposited or provided access to graph files (e.g. BED files) for the called peaks.

Data access links<br>*May remain private before publication.*	https://www.ncbi.nlm.nih.gov/geo/query/acc.cgi?acc=GSE138854
Files in database submission	Primary files (all mouse):<br>GSM4120474 WT rep1 RNA-seq<br>GSM4120475 WT rep2 RNA-seq<br>GSM4120476 WT rep3 RNA-seq<br>GSM4120477 Gulotm1Mae VitC+ rep1 RNA-seq<br>GSM4120478 Gulotm1Mae VitC+ rep2 RNA-seq<br>GSM4120479 Gulotm1Mae VitC+ rep3 RNA-seq<br>GSM4120480 Gulotm1Mae VitC+- rep1 RNA-seq<br>GSM4120481 Gulotm1Mae VitC+- rep2 RNA-seq<br>GSM4120482 Gulotm1Mae VitC+- rep3 RNA-seq<br>GSM4120483 Gulotm1Mae VitC+ rep1 hMeDIP-seq<br>GSM4120484 Gulotm1Mae VitC+ rep2 hMeDIP-seq<br>GSM4120485 Gulotm1Mae VitC+ rep3 hMeDIP-seq<br>GSM4120486 Gulotm1Mae VitC+- rep1 hMeDIP-seq<br>GSM4120487 Gulotm1Mae VitC+- rep2 hMeDIP-seq<br>GSM4120488 Gulotm1Mae VitC+- rep3 hMeDIP-seq<br>GSM4120489 Input DNA Femur<br>GSM4120490 D28_MC3T3-E1 VitC- rep1 hMeDIP-seq<br>GSM4120491 D28_MC3T3-E1 VitC- rep2 hMeDIP-seq<br>GSM4120492 D28_MC3T3-E1 VitC+ rep1 hMeDIP-seq<br>GSM4120493 D28_MC3T3-E1 VitC+ rep2 hMeDIP-seq<br>GSM4120494 Input_DNA_MC3T3<br>GSM5968242 Femur cTet WT rep1 RNA-seq<br>GSM5968243 Femur cTet WT rep2 RNA-seq

GSM5968244 Femur cTet WT rep3 RNA-seq
GSM5968245 Femur cTet1 KO rep1 RNA-seq
GSM5968246 Femur cTet1 KO rep2 RNA-seq
GSM5968247 Femur cTet1 KO rep3 RNA-seq
GSM5968248 Femur cTet2 KO rep1 RNA-seq
GSM5968249 Femur cTet2 KO rep2 RNA-seq
GSM5968250 Femur cTet2 KO rep3 RNA-seq
GSM5968251 Femur cTet1_2 KO rep1 RNA-seq
GSM5968252 Femur cTet1_2 KO rep2 RNA-seq
GSM5968253 Femur cTet1_2 KO rep3 RNA-seq
GSM5968254 Femur cTet WT rep1 hMeDIP-seq
GSM5968255 Femur cTet WT rep2 hMeDIP-seq
GSM5968256 Femur cTet WT rep3 hMeDIP-seq
GSM5968257 Femur cTet1_2 KO rep1 hMeDIP-seq
GSM5968258 Femur cTet1_2 KO rep2 hMeDIP-seq
GSM5968259 Femur cTet1_2 KO rep3 hMeDIP-seq
GSM5968260 Femur Input1
GSM5968261 Femur Input2
GSM5968262 MC3T3-E1 VitC- rep1 H3K9me3 ChIP-seq
GSM5968263 MC3T3-E1 VitC- rep2 H3K9me3 ChIP-seq
GSM5968264 MC3T3-E1 VitC+ rep1 H3K9me3 ChIP-seq
GSM5968265 MC3T3-E1 VitC+ rep2 H3K9me3 ChIP-seq
GSM5968266 MC3T3-E1 VitC- rep1 H3K27me3 ChIP-seq
GSM5968267 MC3T3-E1 VitC- rep2 H3K27me3 ChIP-seq
GSM5968268 MC3T3-E1 VitC+ rep1 H3K27me3 ChIP-seq
GSM5968269 MC3T3-E1 VitC+ rep2 H3K27me3 ChIP-seq
GSM5968270 MC3T3-E1 ChIP_Input_1
GSM5968271 MC3T3-E1 ChIP_Input_2

Processed files:
GSE138854_BigWig_on_WT_hMeDIP-Seq.bigwig
GSE138854_BigWig_on_cT1_2_KO_hMeDIP-Seq.bigwig
GSE138854_D28_MC3T3-E1_VitC+_hMeDIP-seq.bigwig
GSE138854_D28_MC3T3-E1_VitC-_hMeDIP-seq.bigwig
GSE138854_Gulo_VitC+-_hMeDIP-seq.bed.gz
GSE138854_Gulo_VitC+-_hMeDIP-seq.bigwig
GSE138854_Gulo_VitC+_hMeDIP-seq.bed.gz
GSE138854_Gulo_VitC+_hMeDIP-seq.bigwig
GSE138854_MACS2_MC3T3_H3K27me3_VitC_min_Peaks.bed.gz
GSE138854_MACS2_MC3T3_H3K27me3_VitC_pl_Peaks.bed.gz
GSE138854_MACS2_MC3T3_H3K9me3_VitC_min_Peaks.bed.gz
GSE138854_MACS2_MC3T3_H3K9me3_VitC_pl_Peaks.bed.gz
GSE138854_MACS2_callpeak_on_WTs_hMeDIP-Seq.bed.gz
GSE138854_MACS2_callpeak_on_cT1_2_KOs_hMeDIP-Seq.bed.gz
GSE138854_MC3T3-E1_VitC+_hMeDIP-seq.bed.gz
GSE138854_MC3T3-E1_VitC-_hMeDIP-seq.bed.gz
GSE138854_MC3T3_H3K27me3_VitC_min_coverage.tdf
GSE138854_MC3T3_H3K27me3_VitC_pl_coverage.tdf
GSE138854_MC3T3_H3K9me3_VitC_min_coverage.tdf
GSE138854_MC3T3_H3K9me3_VitC_pl_coverage.tdf
GSE138854_Tet_cKOs_RNA_Seq_DeSeq2_normalized_counts_all_samples.txt.gz

Genome browser session
(e.g. UCSC)

No longer applicable

Methodology

Replicates

Number of replicates used for each experiment are outlined in the materials and methods section.

Sequencing depth

Gulotm1Mae VitC+ rep1 hMeDIP-seq, total reads: 53339046, uniquely mapped reads: 48222998, length: 51 bp, paired-end
Gulotm1Mae VitC+ rep2 hMeDIP-seq, total reads: 61838232, uniquely mapped reads: 57080679, length: 51 bp, paired-end
Gulotm1Mae VitC+ rep3 hMeDIP-seq, total reads: 53260303, uniquely mapped reads: 48546567, length: 51 bp, paired-end
Gulotm1Mae VitC+- rep1 hMeDIP-seq, total reads: 62278120, uniquely mapped reads: 58005157, length: 51 bp, paired-end
Gulotm1Mae VitC+- rep2 hMeDIP-seq, total reads: 37348895, uniquely mapped reads: 31264405, length: 51 bp, paired-end
Gulotm1Mae VitC+- rep3 hMeDIP-seq, total reads: 41802384, uniquely mapped reads: 35403491, length: 51 bp, paired-end
Input DNA Femur, total reads: 54225370, uniquely mapped reads: 47620619, length: 51 bp, paired-end
D28_MC3T3-E1 VitC- rep1 hMeDIP-seq, total reads: 62138416, uniquely mapped reads: 55746358, length: 51 bp, paired-end
D28_MC3T3-E1 VitC- rep2 hMeDIP-seq, total reads: 79711613, uniquely mapped reads: 72580102, length: 51 bp, paired-end
D28_MC3T3-E1 VitC+ rep1 hMeDIP-seq, total reads: 66418537, uniquely mapped reads: 63552589, length: 51 bp, paired-end

D28_MC3T3-E1 VitC+ rep2 hMeDIP-seq, total reads: 59469469, uniquely mapped reads: 53193628, length: 51 bp, paired-end
Input_DNA_MC3T3, total reads: 33264885, uniquely mapped reads: 28924373, length: 51 bp, paired-end
Femur cTet WT rep1 hMeDIP-seq, total reads: 52272961, uniquely mapped reads: 48706585, length: 51 bp, paired-end
Femur cTet WT rep2 hMeDIP-seq, total reads: 55132170, uniquely mapped reads: 51711247, length: 51 bp, paired-end
Femur cTet WT rep3 hMeDIP-seq, total reads: 48603568, uniquely mapped reads: 45209782, length: 51 bp, paired-end
Femur cTet1_2 KO rep1 hMeDIP-seq, total reads: 54459031, uniquely mapped reads: 50774439, length: 51 bp, paired-end
Femur cTet1_2 KO rep2 hMeDIP-seq, total reads: 46477281, uniquely mapped reads: 43105276, length: 51 bp, paired-end
Femur cTet1_2 KO rep3 hMeDIP-seq, total reads: 56073761, uniquely mapped reads: 51529801, length: 51 bp, paired-end
Femur Input1, total reads: 77956330, uniquely mapped reads: 67239777, length: 51 bp, paired-end
Femur Input2, total reads: 27105392, uniquely mapped reads: 22218841, length: 51 bp, paired-end
MC3T3-E1 VitC- rep1 H3K9me3 ChIP-seq, total reads: 42747330, uniquely mapped reads: 34925474, length: 51 bp, paired-end
MC3T3-E1 VitC- rep2 H3K9me3 ChIP-seq, total reads: 36284777, uniquely mapped reads: 28276840, length: 51 bp, paired-end
MC3T3-E1 VitC+ rep1 H3K9me3 ChIP-seq, total reads: 45343083, uniquely mapped reads: 34283926, length: 51 bp, paired-end
MC3T3-E1 VitC+ rep2 H3K9me3 ChIP-seq, total reads: 37793264, uniquely mapped reads: 29463276, length: 51 bp, paired-end
MC3T3-E1 VitC- rep1 H3K27me3 ChIP-seq, total reads: 74524318, uniquely mapped reads: 66958813, length: 51 bp, paired-end
MC3T3-E1 VitC- rep2 H3K27me3 ChIP-seq, total reads: 39047559, uniquely mapped reads: 34536804, length: 51 bp, paired-end
MC3T3-E1 VitC+ rep1 H3K27me3 ChIP-seq, total reads: 43225376, uniquely mapped reads: 38654131, length: 51 bp, paired-end
MC3T3-E1 VitC+ rep2 H3K27me3 ChIP-seq, total reads: 40679267, uniquely mapped reads: 36312607, length: 51 bp, paired-end
MC3T3-E1 ChIP_Input_1, total reads: 58834454, uniquely mapped reads: 48826162, length: 51 bp, paired-end
MC3T3-E1 ChIP_Input_2, total reads: 38657243, uniquely mapped reads: 32734099, length: 51 bp, paired-end

Antibodies

5hmC antibody, Active Motif, 39791, 1µg antibody/µg DNA (MeDIP-Seq);
H3K9me3, Diagenode, C15410056, A2810P, 1 µg/IP (ChIP-Seq);
H3K27me3, Cell Signaling Technology, 9733BC, C36B11, 1:50 (ChIP-Seq);

Peak calling parameters

Peaks were defined using MACS2 callpeak Galaxy version 2.1.0.20140616.0 with parameters -g mm and -p 1e-05.

Data quality

Gulotm1Mae VitC+ rep1 hMeDIP-seq, peaks at 5% FDR: 279626, peaks with fold enrichment >5-fold: 120375
Gulotm1Mae VitC+ rep2 hMeDIP-seq, peaks at 5% FDR: 373025, peaks with fold enrichment >5-fold: 158867
Gulotm1Mae VitC+ rep3 hMeDIP-seq, peaks at 5% FDR: 224797, peaks with fold enrichment >5-fold: 91633
Gulotm1Mae VitC+- rep1 hMeDIP-seq, peaks at 5% FDR: 244450, peaks with fold enrichment >5-fold: 72515
Gulotm1Mae VitC+- rep2 hMeDIP-seq, peaks at 5% FDR: 198658, peaks with fold enrichment >5-fold: 107182
Gulotm1Mae VitC+- rep3 hMeDIP-seq, peaks at 5% FDR: 195784, peaks with fold enrichment >5-fold: 94071
D28_MC3T3-E1 VitC- rep1 hMeDIP-seq, peaks at 5% FDR: 283861, peaks with fold enrichment >5-fold: 110758
D28_MC3T3-E1 VitC- rep2 hMeDIP-seq, peaks at 5% FDR: 262548, peaks with fold enrichment >5-fold: 102047
D28_MC3T3-E1 VitC+ rep1 hMeDIP-seq, peaks at 5% FDR: 269821, peaks with fold enrichment >5-fold: 124697
D28_MC3T3-E1 VitC+ rep2 hMeDIP-seq, peaks at 5% FDR: 247393, peaks with fold enrichment >5-fold: 75328
Femur cTet WT rep1 hMeDIP-seq, peaks at 5% FDR: 187543, peaks with fold enrichment >5-fold: 53221
Femur cTet WT rep2 hMeDIP-seq, peaks at 5% FDR: 204529, peaks with fold enrichment >5-fold: 53126
Femur cTet WT rep3 hMeDIP-seq, peaks at 5% FDR: 191335, peaks with fold enrichment >5-fold: 55820
Femur cTet1_2 KO rep1 hMeDIP-seq, peaks at 5% FDR: 176528, peaks with fold enrichment >5-fold: 55739
Femur cTet1_2 KO rep2 hMeDIP-seq, peaks at 5% FDR: 159787, peaks with fold enrichment >5-fold: 49618
Femur cTet1_2 KO rep3 hMeDIP-seq, peaks at 5% FDR: 154973, peaks with fold enrichment >5-fold: 44409
MC3T3-E1 VitC- rep1 H3K9me3 ChIP-seq, peaks at 5% FDR: 54798, peaks with fold enrichment >5-fold: 10662
MC3T3-E1 VitC- rep2 H3K9me3 ChIP-seq, peaks at 5% FDR: 12772, peaks with fold enrichment >5-fold: 3133
MC3T3-E1 VitC+ rep1 H3K9me3 ChIP-seq, peaks at 5% FDR: 52897, peaks with fold enrichment >5-fold: 16053
MC3T3-E1 VitC+ rep2 H3K9me3 ChIP-seq, peaks at 5% FDR: 12115, peaks with fold enrichment >5-fold: 4933
MC3T3-E1 VitC- rep1 H3K27me3 ChIP-seq, peaks at 5% FDR: 107119, peaks with fold enrichment >5-fold: 303
MC3T3-E1 VitC- rep2 H3K27me3 ChIP-seq, peaks at 5% FDR: 62428, peaks with fold enrichment >5-fold: 4
MC3T3-E1 VitC+ rep1 H3K27me3 ChIP-seq, peaks at 5% FDR: 65906, peaks with fold enrichment >5-fold: 44
MC3T3-E1 VitC+ rep2 H3K27me3 ChIP-seq, peaks at 5% FDR: 60097, peaks with fold enrichment >5-fold: 15

Software

Fastq files from MeDIP-Seq and ChIP-seq experiments were assessed for run quality using fastqc version 0.72. Mapping to reference genome mm10 was performed using BWA Galaxy version 0.7.15.1 using standard settings. Peak calling was performed with MACS2 callpeak Galaxy version 2.1.0.20140616.0 using -g mm and -p 1e-05 and differential peak analysis with DiffBind Galaxy version 2.6.6.4 or 2.10.0. Genomic 5hmC and ChIP-Seq related peak distribution was assessed with CEAS software (Version1.0.0, Cistrome, Liu Lab). Super-enhancer assessment was performed by using the package ROSE as integrated in the software NaviSE (Ascension M e al., BMC Bioinformatics 2017) by stitch neighboring peaks within 12.5kb from each other were stitched together while excluding regions ± 2000 bps from any transcription start site. Overlapping SE between samples were assessed with Bedtools (version 2.29). MeDIP-Seq and ChIP-Seq based gene-ontology was performed using GREAT version 3.0.0 (Bejerano Lab, Stanford; http://great.stanford.edu/public/html/).
