# Supplementary material for: Vitamin C epigenetically controls osteogenesis and bone mineralization
Source: Nat Commun. 2022 Oct 6;13:5883. doi: 10.1038/s41467-022-32915-8 (PMC9537512; doi:10.1038/s41467-022-32915-8)
Supplement: Supplementary file 4 — Source Data [file 41467_2022_32915_MOESM4_ESM.zip › Uncropped Western Blot Images for Main Figures.pptx]

## Slide 1
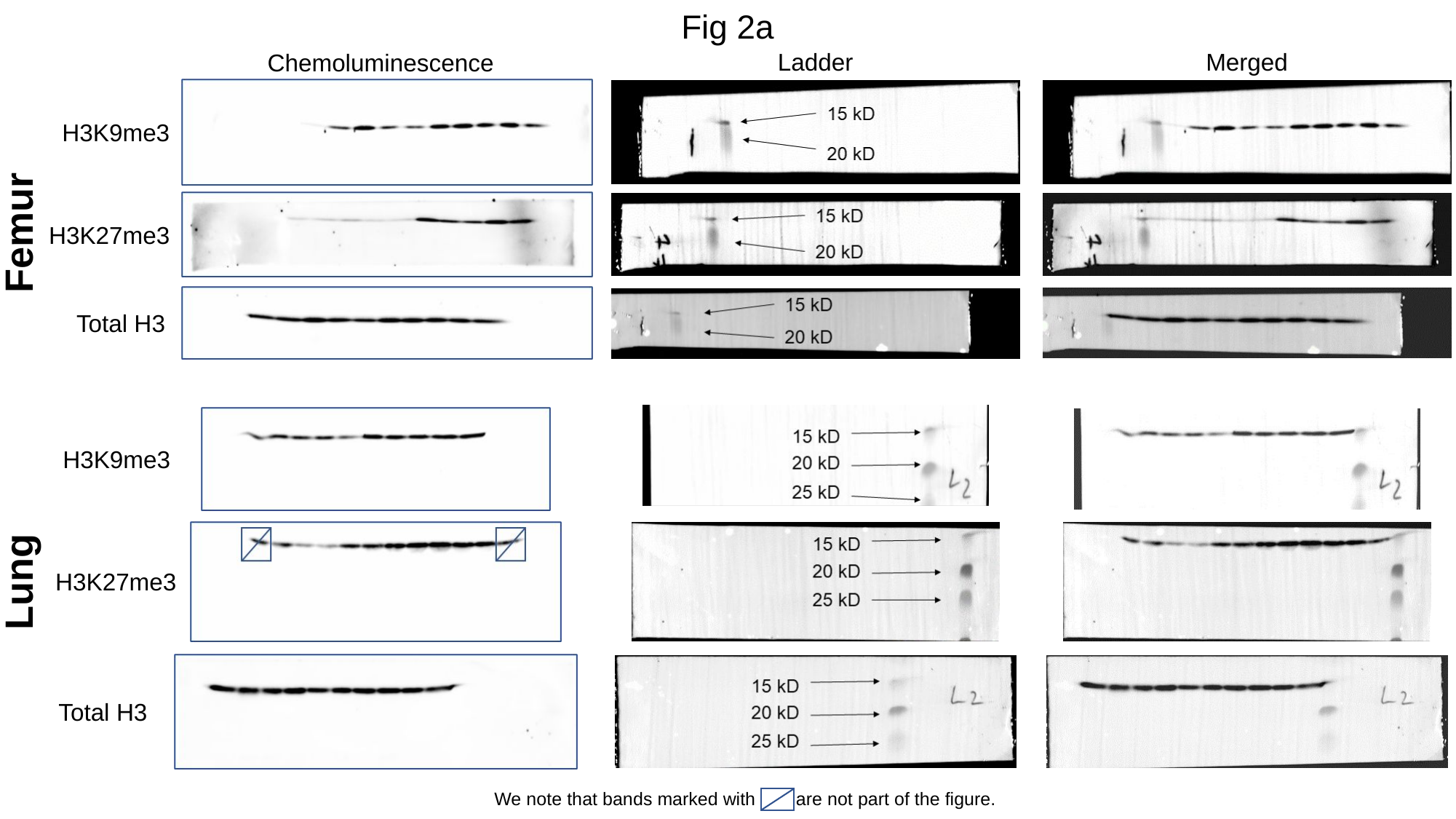

Fig 2a
Merged
Ladder
Chemoluminescence
H3K9me3
Femur
H3K27me3
Total H3
H3K9me3
Lung
H3K27me3
Total H3
We note that bands marked with are not part of the figure.

## Slide 2
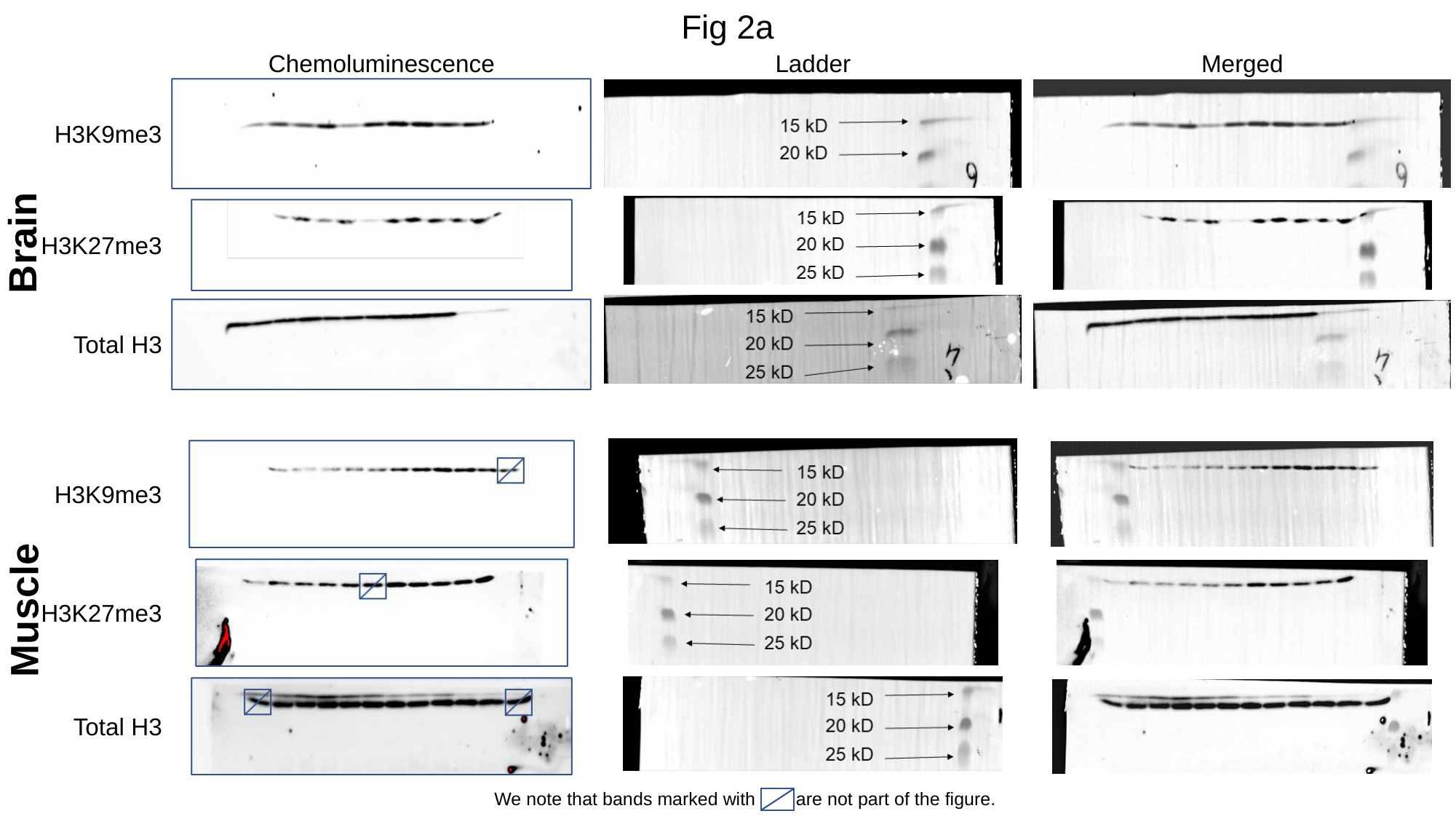

Fig 2a
Merged
Ladder
Chemoluminescence
H3K9me3
Brain
H3K27me3
Total H3
H3K9me3
Muscle
H3K27me3
Total H3
We note that bands marked with are not part of the figure.

## Slide 3
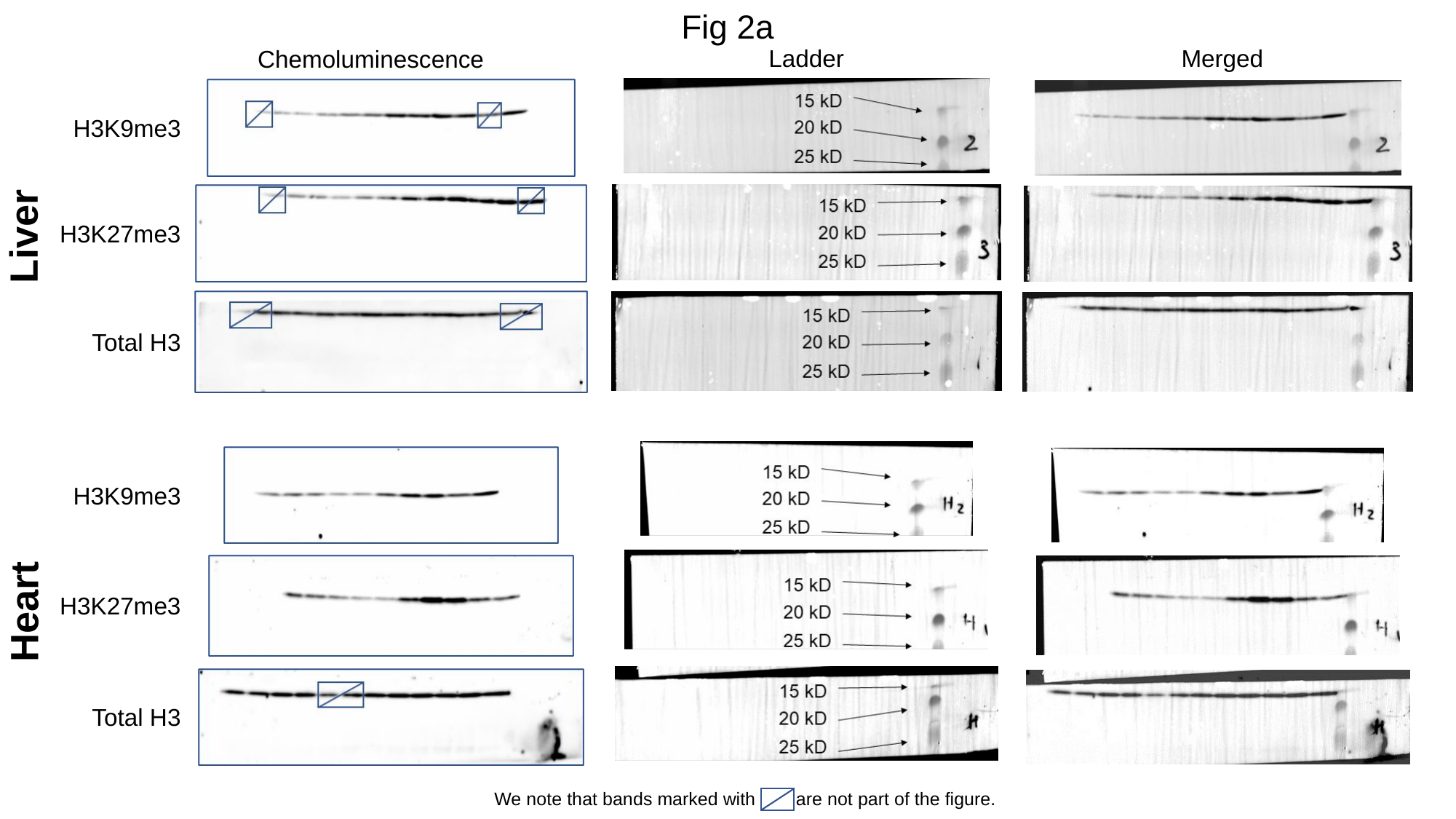

Fig 2a
Merged
Ladder
Chemoluminescence
H3K9me3
Liver
H3K27me3
Total H3
H3K9me3
Heart
H3K27me3
Total H3
We note that bands marked with are not part of the figure.

## Slide 4
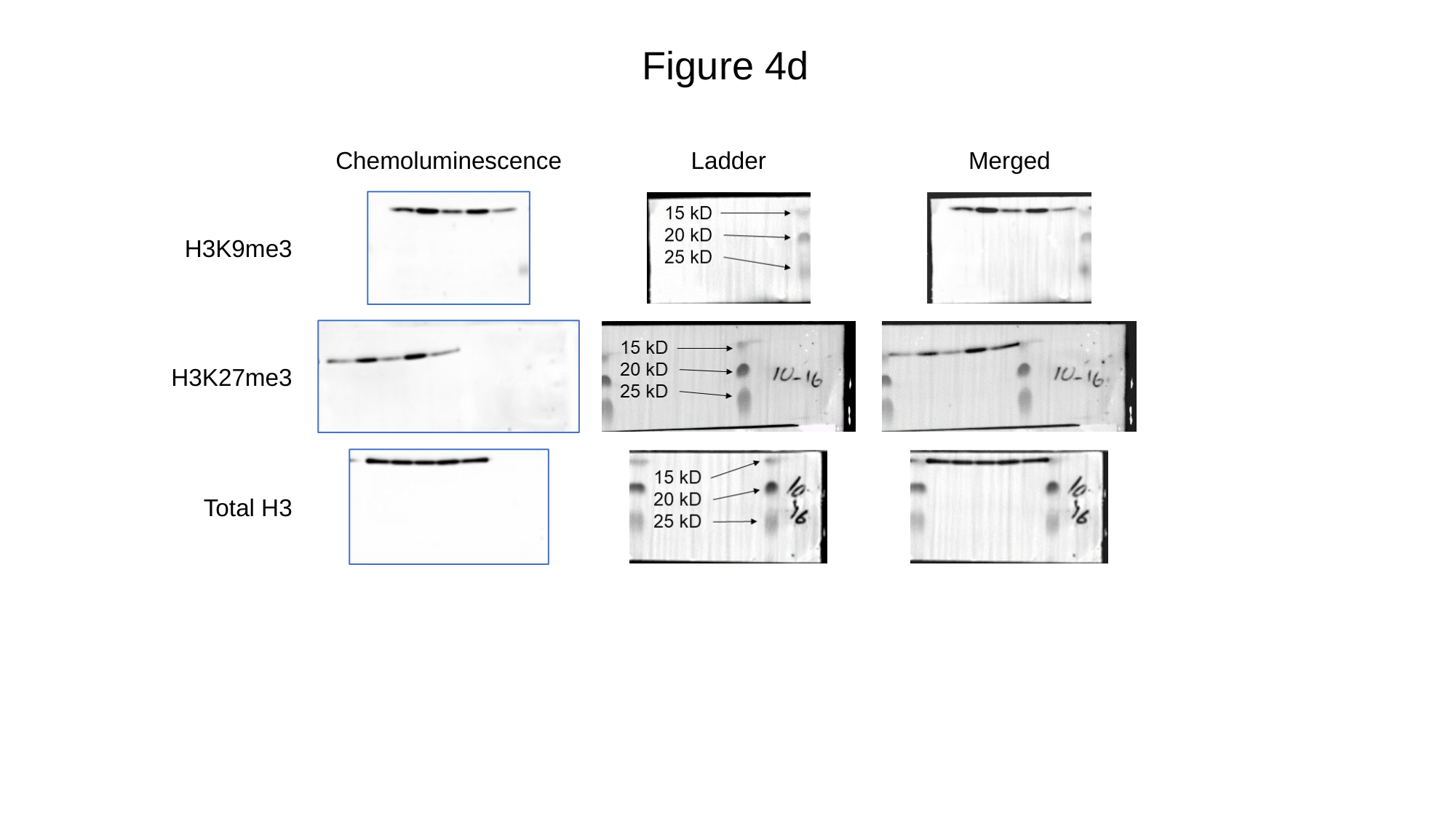

Figure 4d
Chemoluminescence
Ladder
Merged
H3K9me3
H3K27me3
Total H3

## Slide 5
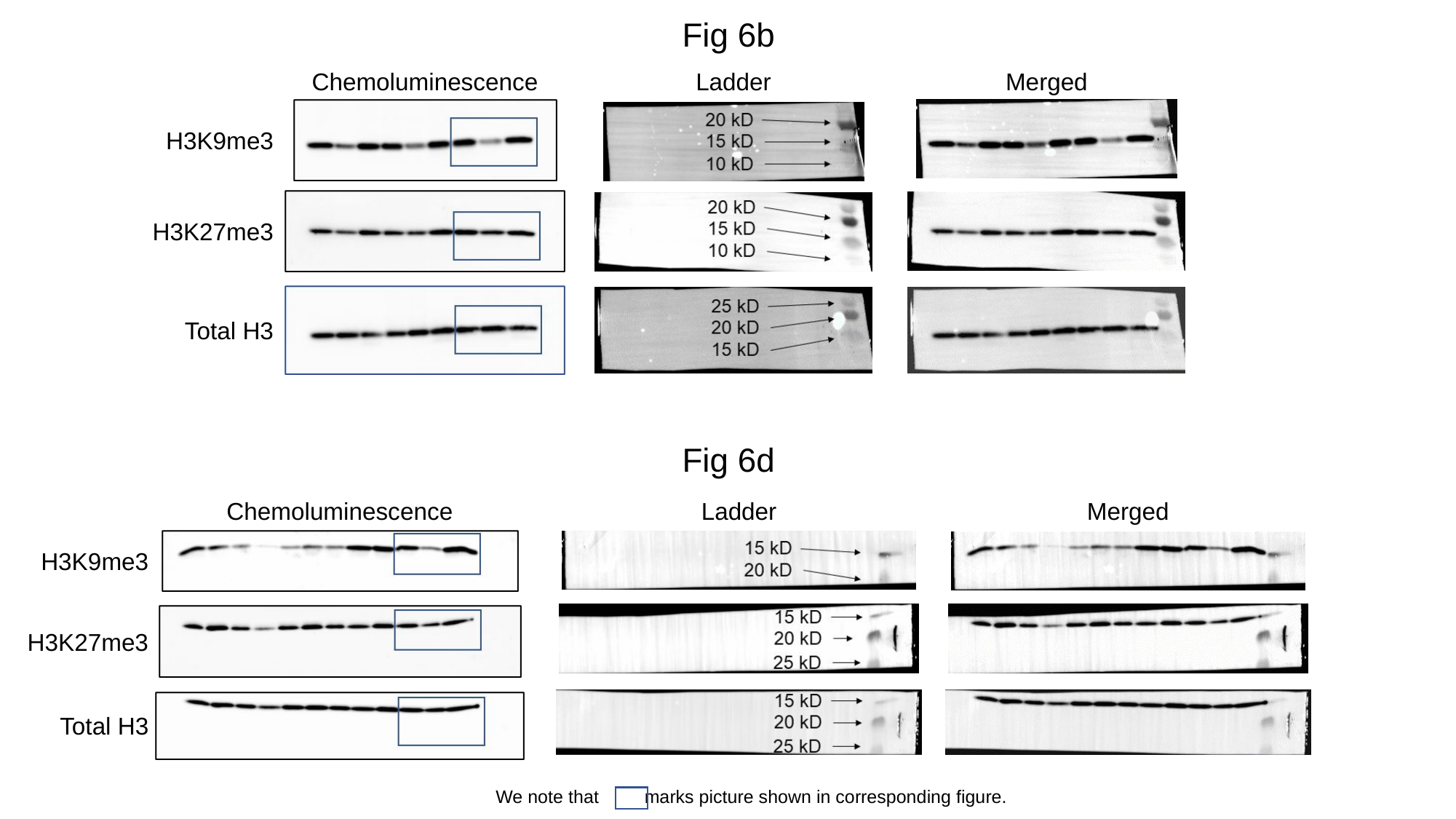

Fig 6b
Chemoluminescence
Ladder
Merged
H3K9me3
H3K27me3
Total H3
Fig 6d
Chemoluminescence
Ladder
Merged
H3K9me3
H3K27me3
Total H3
We note that marks picture shown in corresponding figure.

## Slide 6
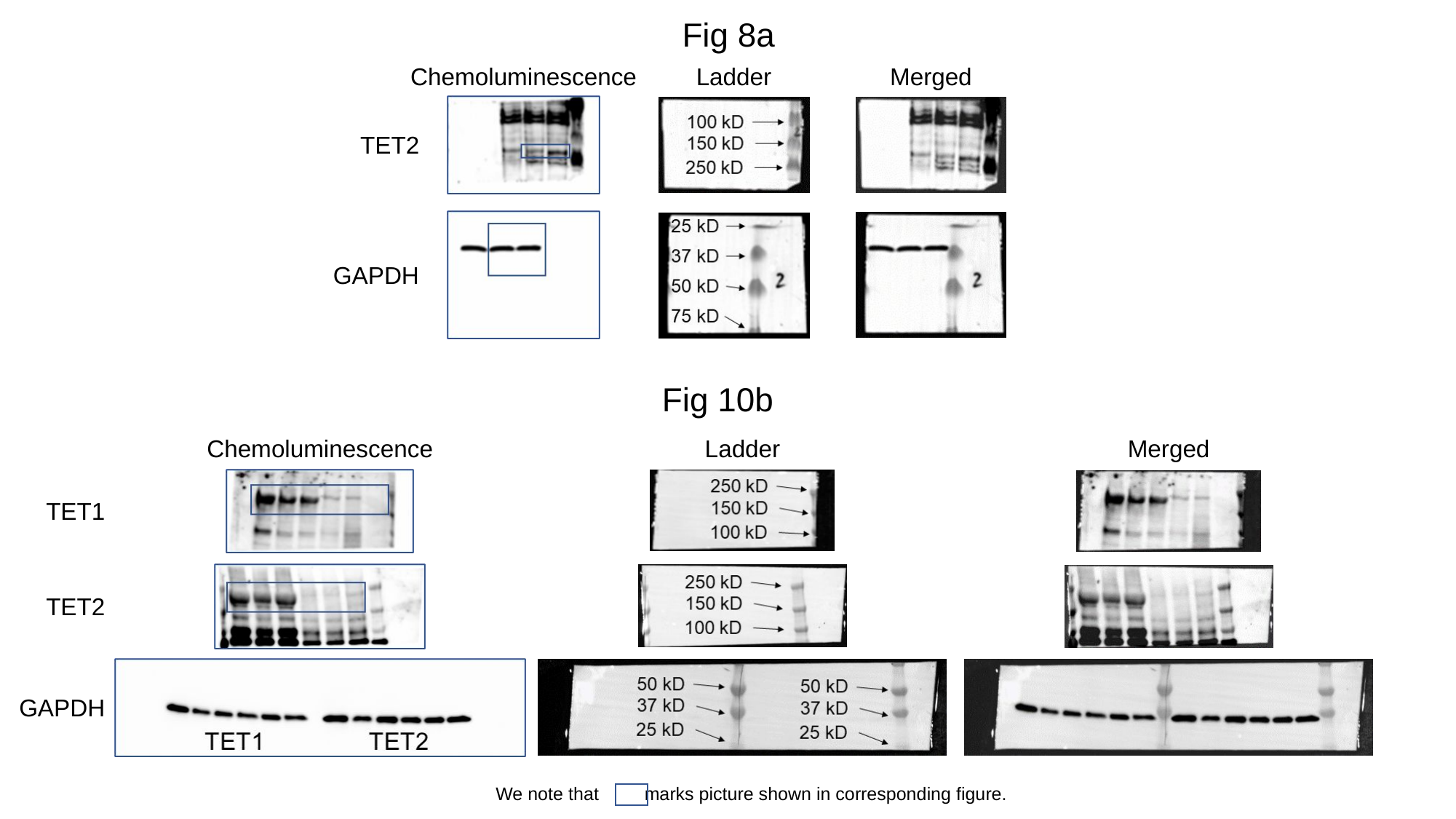

Fig 8a
Chemoluminescence
Ladder
Merged
TET2
GAPDH
Fig 10b
Chemoluminescence
Ladder
Merged
TET1
TET2
GAPDH
We note that marks picture shown in corresponding figure.
